# Effects of interventions for social anxiety and shyness in school-aged children: A systematic review and meta-analysis

**DOI:** 10.1371/journal.pone.0254117

**Published:** 2021-07-09

**Authors:** Reinie Cordier, Renée Speyer, Natasha Mahoney, Anne Arnesen, Liv Heidi Mjelve, Geir Nyborg

**Affiliations:** 1 Department of Social Work, Education and Community Wellbeing, Northumbria University, Newcastle upon Tyne, United Kingdom; 2 Faculty of Health Sciences, School of Occupational Therapy, Social Work and Speech Pathology, Curtin University, Perth, Australia; 3 Department of Special Needs Education, University of Oslo, Oslo, Norway; 4 Department of Otorhinolaryngology and Head and Neck Surgery, Leiden University Medical Centre, Leiden, Netherlands; 5 The Norwegian Center for Child Behavioral Development (NUBU), Oslo, Norway; Temple University, UNITED STATES

## Abstract

In school, shyness is associated with psychosocial difficulties and has negative impacts on children’s academic performance and wellbeing. Even though there are different strategies and interventions to help children deal with shyness, there is currently no comprehensive systematic review of available interventions. This systematic review and meta-analysis aim to identify interventions for shy children and to evaluate the effectiveness in reducing psychosocial difficulties and other impacts. The methodology and reporting were guided by the Preferred Reporting Items for Systematic Reviews and Meta-Analyses statement and checklist. A total of 4,864 studies were identified and 25 of these met the inclusion criteria. These studies employed interventions that were directed at school-aged children between six and twelve years of age and described both pre- and post-intervention measurement in target populations of at least five children. Most studies included an intervention undertaken in a school setting. The meta-analysis revealed interventions showing a large effect in reducing negative consequences of shyness, which is consistent with extant literature regarding shyness in school, suggesting school-age as an ideal developmental stage to target shyness. None of the interventions were delivered in a classroom setting, limiting the ability to make comparisons between in-class interventions and those delivered outside the classroom, but highlighting the effectiveness of interventions outside the classroom. The interventions were often conducted in group sessions, based at the school, and involved activities such as play, modelling and reinforcement and clinical methods such as social skills training, psychoeducation, and exposure. Traditionally, such methods have been confined to a clinic setting. The results of the current study show that, when such methods are used in a school-based setting and involve peers, the results can be effective in reducing negative effects of shyness. This is consistent with recommendations that interventions be age-appropriate, consider social development and utilise wide, school-based programs that address all students.

## Introduction

Shyness is commonly experienced by school-aged children [1]. Despite being a frequently used term, there is a diversity of constructs that underpin ‘shyness’, including behavioural inhibition, social reticence, social withdrawal, anxious solitude and social anxiety [2]. There have been several approaches to defining shyness in the past. Some conceptualisations theorise shyness as either behavioural inhibition to the unfamiliar (i.e., wariness in unfamiliar situations) or social withdrawal [i.e., elevated rates of solitary behaviour or symptoms of social anxiety disorder; 3–7]. In contrast, substantial literature has investigated shyness as encompassing individual differences in wariness or anxiety in novel situations, embarrassment or self-conscious in anticipation of social evaluation and reticence in social situations [7]. Shyness has also been considered from a developmental perspective, proposing an interactional child-by-environment model. By this model, behavioural inhibition and social withdrawal are considered risk factors for further social anxiety. Interactions between the child and the environment, and the child and their parents and peers, can either promote or diminish the risk of later anxiety [4,8,9].

### Taxonomy of shyness

In order to organise and operationalise the various concepts of shyness in use, Rubin, Coplan [7] proposed a taxonomy of shyness. This taxonomy places behavioural solitude (i.e., lack of interaction in presence of peers) as the over-arching, observable behaviour of shyness. The source of this solitude is either internal, termed *social withdrawal* (i.e., removing oneself from social interaction) or external, termed *active isolation* (i.e., being excluded by others). If the source is internal (i.e., social withdrawal), the motivation for withdrawal is either by preference, termed *social disinterest*, or a result of *fear or wariness*. The source of fear is then split into four categories: 1) b*ehaviour inhibition* (i.e., fear of novelty); 2) *anxious solitude* (i.e., wariness in familiar social situations); 3) *shyness* (i.e., wariness of social novelty and/or perceive evaluation); and 4) *social reticence* (i.e., observed display of onlooker behaviours). In this taxonomy, these fears and behaviours can become clinically significant over time and manifest as a social anxiety disorder. This taxonomy provides a clear conceptualisation of shyness and social anxiety, and outlines observable behaviours, sources, motivations and specific fears.

### Shy children in school

In addition to the potential manifestation of social anxiety disorder theorised by Rubin, Coplan [7], children with shyness may also experience a range of other difficulties that, although not clinically diagnosable, can vastly impact their wellbeing, social networks and academic performance [10]. Many of these difficulties are experienced at school, where peer interactions are an integral component of the environment. Shy children are often quiet across a range of situations in school, both in the classroom and in social situations [11]. Talking, in or outside of class, can make a child the centre of attention and open to social evaluation, which sits at the centre of the taxonomy of shyness. Shy children have fewer in-class interactions and respond less often to direct or class-wide questions than their non-shy peers [12]. Research has shown that shy children often have lower academic attainment, poorer performance on tests of language development, and are more likely to have difficulty adjusting at school [10].

Shyness is also associated with psychosocial challenges in school. Shy children often have a limited number of friends and are at risk of peer victimisation and exclusion [7,13]. They may also use social withdrawal as a way to avoid or cope with peer victimisation [14]. Shyness is positively associated with somatic complaints, school-related stress, anxiety and depressive symptoms [15,16]. Shyness can increase over time, predicting difficulties later in adolescence [17]. Shy children often have poor social skills and high levels of anxiety and depression symptoms in early adolescence [17]. Longitudinal studies show that shyness and social withdrawal are significant risk factors for social anxiety disorder [8,18]. These results are aligned with the Rubin, Coplan [7] taxonomy of shyness and social anxiety, demonstrating the theorised pathway to social anxiety disorder.

### School-based interventions for shy children

Given the short- and long-term psychosocial and academic outcomes for shy children, there have been multiple attempts at buffering the impacts of shyness. In the classroom, teachers can use concepts, such as shyness, as a tool to tailor how they work with an individual child [19]. Teachers at a Norwegian elementary school broadly categorised shy children in their classroom as either, 1) withdrawn, 2) anxious, and/or 3) having poor self-esteem. These categories then informed the support given to the individual child, including cognitive support and feedback and encouraging active learning [19]. Informal, teacher-facilitated support or intervention is a common response to shyness within the classroom, as teachers recognise shy children and the potential problems they encounter [20–22]. Teachers report employing social learning strategies, such as verbal encouragement, praise and modelling behaviour, as well as peer-focused strategies to promote inclusion, such as encouraging joint activities [20]. However, the effectiveness of these individual attempts is limited to within the classroom and may not impact poor psychosocial outcomes for shy children in broader contexts.

Beyond classroom support, there are many different structured interventions targeting shyness in school-aged children. Clinical interventions are typically conducted in non-naturalistic settings with homework-style practice in naturalistic settings, and comprise of social skills training, psychoeducation, cognitive restructuring and exposure tasks [8]. Criticisms of this approach are that such interventions do not consider nor change the environment itself and focus on treating social anxiety disorders, ignoring shyness more broadly [8]. Clinical interventions need to be age-appropriate and consider cognitive and social development, social context and parent involvement [23]. As shy children are often excluded or victimised by their peers, interventions need to consider the environment and peer interaction. Developmental interventions include peers in the intervention itself, aiming to increase the use of successful social skills in naturalistic settings [8]. However, this approach requires school resources and willingness of peers to be involved. Crozier [1] suggests that a focus on individual screening and pathologising shyness may not lead to effective intervention, as not all shy children develop anxiety disorders. Wider, school-based programs that address all student’s social confidence, instead of targeted interventions, may be more suitable intervention for shyness [1]. Given the wide range of intervention approaches and intervention programs themselves, there is no clear best-practice for interventions for shy children. This is further complicated by inconsistent use of terminology related to shyness [1].

To reduce academic and concomitant psychosocial difficulties in school for shy children, there is a need for effective, feasible interventions. To date, there is no comprehensive systematic review of the available interventions for shy children. This systematic review and meta-analysis aim to provide an overview of the available interventions for shy children aged six to twelve years, describe the characteristics of the interventions, summarise intervention strategies being used, and determine their overall effectiveness, as well as effectiveness of interventions in relation to the following domains: 1) setting where the interventions is delivered; 2) mode of delivery; 3) intervention focus; and 4) rater of outcome measures.

## Method

The methodology and reporting on this systematic review were guided by the Preferred Reporting Items for Systematic Reviews and Meta-Analyses (PRISMA) statement and checklist. The PRISMA statement and checklist supports researchers in the critical and transparent reporting of systematic reviews in areas of health care [24,25].

[The PRISMA checklist is provided as Supporting Information].

### Eligibility criteria

To be eligible for inclusion in this systematic review, studies were required to describe an intervention in school-aged children (between six and twelve years old) for social anxiety and shyness. Only studies describing both pre- and post-intervention measurement in target populations of at least five children were included. Only original articles published in English were considered for eligibility. Conference abstracts, case reports, reviews, student dissertations and editorials were excluded.

### Data sources and search strategies

Literature searches were conducted in five electronic databases: CINAHL, Embase, Eric, PsycINFO and PubMed. All publication dates up to 23^rd^ December 2020 were included. The search strategies per database are listed in Table 1.

**Table 1:** Search strategies per literature database.

Database and search terms (subject headings and free text words)
**CINAHL**: ((MH "Shyness") OR (MH "Social Isolation") OR (MH "Social Isolation (Saba CCC)") OR (MH "Impaired Social Interaction (NANDA)") OR (MH "Social Isolation (NANDA)")) AND ((MH "Clinical Effectiveness") OR (MH "Treatment Outcomes") OR (MH "Effect Size") OR (MH "Outcome Assessment") OR (MH "Outcomes (Health Care)+") OR (MH "Intervention Trials") OR (MH "Program Evaluation") OR (MH "Evaluation+") OR (MH "Course Evaluation") OR (MH "Evaluation Research+"))
**Embase**: (shyness/ OR introversion/ OR psychosocial withdrawal/ OR loneliness/ OR social isolation/ OR internalization/) AND (treatment outcome/ OR measurement/ OR intervention study/ OR program evaluation/ OR program effectiveness/ OR program efficacy/ OR evaluation research/ OR evaluation study/ OR course evaluation/)
**Eric**: (shyness/ OR extraversion introversion/ OR "withdrawal (psychology)"/ OR Social isolation/) AND (effect size/ OR efficiency/ OR outcome measures/ OR treatment duration/ OR treatment outcome/ OR treatment response/ OR measurement/ OR intervention/ OR program administration/ OR program effectiveness/ OR program evaluation/ OR evaluation/ OR evaluation research/ OR course evaluation/ OR courses/ OR "outcomes of treatment"/ OR efficiency/)
**PsycINFO**: (timidity/ OR introversion/ OR social anxiety/ OR "inhibition (personality)"/ OR loneliness/ OR social isolation/ OR timidity/ OR approach avoidance/ OR internalization/) AND ("effect size (statistical)"/ OR Efficiency OR intervention/ OR program evaluation/ OR treatment/ OR evaluation/ OR course evaluation/)
**PubMed**: ("Shyness"[Mesh] OR "Introversion (Psychology)"[Mesh] OR "Inhibition (Psychology)"[Mesh] OR "Loneliness"[Mesh] OR "Social Isolation"[Mesh] OR "Social Communication Disorder"[Mesh] OR "Adjustment Disorders"[Mesh] OR "Emotional Adjustment"[Mesh]) AND ("Treatment Outcome"[Mesh] OR "Program Evaluation"[Mesh] OR "Outcome Assessment (Health Care)"[Mesh] OR "Outcome and Process Assessment (Health Care)"[Mesh] OR "Patient Outcome Assessment"[Mesh] OR "Self-Evaluation Programs"[Mesh] OR "Efficiency"[Mesh])

### Methodological quality and level of evidence

The Qualsyst critical appraisal tool by Kmet [26] and the National Health and Medical Research Council (NHMRC) Evidence Hierarchy Levels of Evidence [27] were used to assess the methodological quality of the included studies: I (systematic review of level II studies); II (randomised controlled trial); III-1 (pseudo-randomised controlled trial); III-2 (comparative study with concurrent controls); III-3 (comparative study without concurrent controls); IV (case series with either post-test or pre-post outcomes). The Qualsyst tool provides a systematic, reproducible and quantitative means of appraising the methodological quality of research across a broad range of study designs. The Qualsyst consists of 14 items. All items have a three-point ordinal scoring (yes = 2, partial = 1, no = 0). A total score can be converted into a percentage score. A score above 80% is considered strong quality, a score of 60 to 79% considered good, a score of 50 to 59% considered adequate, and a score below 50% considered poor quality. Studies with poor study quality were excluded from further analysis in this review.

### Data extraction

A data extraction form was created to extract data from the included studies under the following categories: study design (according to NHMRC level), methodological quality (Qualsyst), participants (numbers, groups), age (range, mean, standard deviation), gender, intervention, inclusion criteria of the individual study (if stated), outcome measures and treatment outcomes. To ensure the meta-analysis focused on factors that impact on shyness, authors identified and extracted only data collected using the main outcome measure related to shyness (see Table 2). Due to the lack of dedicated shyness outcome measures in literature, the most suitable outcome measure related to shyness was chosen. Data including means, standard deviations, and sample sizes were extracted from the included studies to enable the calculation of the overall effect of shyness interventions (within-group pre-post intervention comparisons), and comparisons between shy children and control groups (between-group experimental vs. control intervention group comparisons).

**Table 2:** Characteristics of included studies.

Treatment/Target skills	Reference/Location	Study Design^1^ and Quality^2^	Participant groups	Inclusion/Exclusion/Shyness Definition	Shyness Outcome Measure	Treatment Outcome
**Social Effectiveness Training for Children (SET-C)** Social skills, anxiety, fear, interpersonal functioning, participation in social activities	Beidel, Turner [30],USA	**Design** III-1	**Total sample**: *N* = 50 *Age*: Not reported *Gender*: Not reported *Diagnosis* (N): social phobia (50), panic disorder (1), generalised anxiety (5), specific phobia (3), OCD (2), separation anxiety (4), adjustment disorder (1), selective mutism (4), ADHD (8)20	**Inclusion**: Primary diagnosis of social phobia and/or social fears at a subclinical level	**Self-report** ● Eysenck Personal Inventory ● SPAI-C+ ● STAI-C ● Loneliness Scale ● Daily Diary of stressful events	Significant effect on extroversion, total social anxiety and phobia scores, K-GAS severity, ADIS-C severity, loneliness, state and trait anxiety, neuroticism, internalising behaviours and play skills for treatment group (*p* < .05)
		**Quality** Strong 88% (21/24)	**Intervention:** *N* = 30 *Age*: 10.5 ± 1.6 *Gender*: 47% *M*, 53% *F* *Diagnosis*: Not reported	**Exclusion:** None reported	**Parent report** ● CBCL	67% of treatment group no longer met diagnostic criteria for social phobia
			**Control:** *N* = 20 *Age*:10.6±1.4 *Gender*: 30% *M*, 70% *F* *Diagnosis*: Not reported	**Definition:** Social phobias, fears of interpersonal interactions and public performances	**Teacher report** NA **Clinician rating** ● K-GAS ● ADIS-C	Non-significant tread for read-aloud effectiveness (*p* < .07)
					**Observations** ● Behavioural assessment during role-play	Improvements maintained at 6-month follow up
	Beidel, Turner [31], USA	**NHMRC Level** III-1	**Total sample:** *N = 122* *Age*: 11.61±2.6 *Gender*: 53.3% *M*, 46.7% *F* *Diagnosi*s (%): Social phobia (100*)*, generalised anxiety (31), specific phobia (14), separation anxiety (11), dysthymic disorder (4.1), selective mutism (10), ADHD (12), language/reading disorder (0.8), learning disorder NOS (0.8)	**Inclusion**: ages 7 to 17, primary diagnosis of social phobia	**Self-report** ● MASC ● SPAI-C+ ● Loneliness Scale ● Daily Diary of stressful	53% of treatment group no longer met diagnostic criteria (*p* < .001)
		**Quality** Strong 88% (21/24)	**Intervention:** *N* = 57 *Age*: Not reported *Gender*: Not reported *Diagnosis*: Not reported	**Exclusion:** Co-existing disorder with higher severity rating than primary, co-morbid bipolar disorder, psychosis, conduct disorder, autism spectrum disorders and intellectual disability; active suicidal ideation; previous unsuccessful trial of fluoxetine or behaviour therapy	**Parent report** ● CBCL	Significant reduction in severity of social phobia between treatment and placebo (*p* < .05); non-sig between treatment and placebo Significant reduction in behavioural avoidance for treatment group (*p* < .05)
			**Fluoxetine:** *N* = 33 *Age*: Not reported *Gender*: Not reported *Diagnosis*: Not reported	**Definition**: Social phobias, fears of interpersonal interactions and public performances	**Teacher report** NA	Significant improvement in social skills and anxiety Non-significant difference in observer rating of anxiety (*p* < .05)
			**Placebo:** *N* = 32 *Age*: Not reported *Gender*: Not reported *Diagnosis*: Not reported		**Clinician rating** ● K-GAS ● CGI ● ADIS-C	All treatment gains maintained at 12-month follow-up
					**Observations**-Behavioural assessment during role-play	
	
**Problem-solving and conversational skills training** Recognising a problem, defining a problem, generating solutions, evaluating consequences, determining best solution, implementing a solution, listening, talking about oneself, initiating conversations, making requests of others	Christoff, Scott [32], USA	**NHMRC Level** III-3	**Total sample:** *N = 6* *Age*: $\bar{x}$ 12.8, 12–14 *Gender*: 2 *M*, 4 *F* *Diagnosis*: Typically developing	**Inclusion**: Recommendation by school staff; appear to lack skills for effectively socialising with peers, few friends, did not attend extracurricular events, appeared to be “loners”	**Self-report** ● Conversation diary of preceding 24 hr period ● Self-Esteem Scale ● Social Interaction Survey ● Self rating of academic performance, ability to get along with others, number of friends, ability to converse, comfort talking to others, number of extracurriculars	Problem-solving effectiveness increased above baseline levels, immediately after introduction of problem-solving training
		**Quality** Good 77% (17/22)		**Exclusion:** None reported		Conversation skills increase on first two baseline assessments; then decreased on third and fourth baseline assessments
				**Definition:** Not reported	**Parent report** ● Subject rating of academic performance, ability to get along with others, number of friends, ability to converse, comfort talking to others, number of extracurriculars	Introduction of problem-solving training lead to increase to specific conversational skills, above baseline levels
						Introduction of conversational skills training led to increases in conversational skills, effective behaviour and overall conversational qualit
					**Teacher report** ● Subject rating of academic performance, ability to get along with others, number of friends, ability to converse, comfort talking to others, number of extracurriculars	Quality ratings and number of appropriate statements increased over time
						Question-asking skills showed less change over time
					**Clinician ratings** ● Problem-solving effectiveness, based on means-end problem-solving ● audio of peer-peer conversations (specific skills, effective behaviour, overall quality) ● Cafeteria observations+	Significant interaction between interaction frequency and higher self-esteem
					**Observations** NA	Significant increase in social interaction scores
						Significant increase in mean ratings of social adjustment, conversational ability and extracurriculars
**Turtle Program** Social skill, introducing self, eye contact, communication, relaxation, expressing emotions, working together, exposure to fear	Chronis-Tuscano, Rubin [33], USA	**NHMRC Level** III-2	**Total Sample:** *N* = 41 *Age*: Not reported *Gender*: Not reported *Diagnosis*: Not reported	**Inclusion:** 42 to 60 months, Behavioural Inhibition Questionnaire > 132	**Self report** NA	Significant Time x Group interactions for anxiety symptoms, favouring treatment group
		**Quality** Strong 93% (26/28)	**Treatment:** *N =* 18 *Age*: 50.81± 9.37 months *Gender*: 50% *M*, 50% *F* *Diagnosis* (%): Social phobia (72), any anxiety disorder (77.8), selective mutism (11.1), specific phobia (5.5), separation anxiety (16.7), major depressive disorder (11.1), ADHD (5.5), ODD (5.5)	**Exclusion:** Social Communication Questionnaire score > 15	**Parent report** ● Preschool Age Psychiatric Assessment + ● BIQ ● CBCL ● PAS; Total and social anxiety scales	Treatment effects on social anxiety marginally significant, medium effect size
			**Waitlist:** *N* = 22 *Age*: 54.27 ± 10.19 *Gender*: 36% *M*, 64% *F* *Diagnosis* (%): social phobia (45), any anxiety disorder (45), specific phobia (4.5), separation anxiety (4.5), major depressive disorder (4.5)	**Definition:** behavioural inhibition, social reticent behaviours	**Teacher report** ● SAS; Total and social anxiety scales	Significant Time x Group interactions on BIQ, CBCL Internalising scale, PAS social anxiety scale, greater improvements in treatment group
					**Clinician rating** NA	Teachers reported significant reductions for treatment group in total and generalised anxiety with medium to large effect size, compared to waitlist
					**Observations** ● Positive Affect/Sensitivity and Negative Control of parent during free play with child	Significant Time x Group interaction on maternal Affect/Sensitivity during free play, greater improvement in treatment group with medium effect size
						No treatment effects on maternal Negative Control
**The Courage and Confidence Mentor Program** Internalising problems	Cook, Xie (30), USA	**NHMRC Level** IV	**Total sample:** *N* = 5 *Age*: 6^th^ to 8^th^ grade (11–14 years) *Gender*: 3 *M*, 2 *F* *Diagnosis*: Typically developing	**Inclusion:** SIBS score > 8, < 15; SUD ratings > 6 across two consecutive days	**Self report** ● SUD ● CIRP	Teachers reported intervention to be reasonable, acceptable and effective Students found intervention acceptable on CIRP
		**Quality** Strong 82% (18/22)		**Exclusion:** None reported	**Parent report** NA	SUD ratings of all participants decreased from baseline (*M* = 7.3) to end of intervention (*M* = 3.3).
				**Definition:** Internalising problems	**Teacher report** ● SIBS ● TRF; Internalising Scale+ ● Intervention Rating Profile	
					**Clinician rating** NA	
					**Observations** NA	
**Play Skills for Shy Children** Social skills, initiating and maintaining interactions, expressing and understand emotions, relaxation techniques	Coplan, Schneider [35], Canada	**NHMRC Level** II	**Total sample:** *N* = 22 *Age*: 56.25±5.99 months *Gender*: 11 *M*, 11 *F* *Diagnosis*: Typically developing	**Inclusion:** between 48 and 60 months of age, parent-rating BIQ scores above top 15% cut-off, SDQ scores below borderline range for conduct and hyperactivity-inattention, child and one parent willing to participant	**Self report** NA	Children in intervention group displayed significantly less reticent-wary behaviours during free-play, compared to waitlist
		**Quality** Strong 86% (24/28)	**Intervention:** *N* = 11 *Age*: Not reported *Gender*: 7 *M*, 4 *F* *Diagnosis*: Typically developing	**Exclusion**: None reported	**Parent report** ● BIQ ● SDQ	Children in intervention group displayed significantly more socially competent behaviours during free-play, compared to waitlist
			**Waitlist Control:** *N* = 11 *Age*: Not reported *Gender*: 4 *M*, 7 *F* *Diagnosis*: Typically developing	**Definition:** behavioural inhibition, wary and reticent behaviours during novel settings with unfamiliar adults or peers	**Teacher report** ● CBS	No significant effect of teacher-rated anxious behaviours or prosocial behaviours
					**Clinician rating** NA	
					**Observations** ● Behaviours during free-play+	
Emotion regulation and awareness, psychosomatic complaints	Fiat, Cook [34], USA	**NHMRC Level** III-2	**Total sample:** *N* = 6 *Age*: $\bar{x}$ 8.9, 7–10 years *Gender*: 3 M, 3 F *Diagnosis* (%): Specific learning disability (33)	**Inclusion:** SIBS score > 8, < 15; SUD ratings > 6 across two consecutive days	**Self report** ● SUD ● CIRP	All but one participant showed reduction in subjective distress
		**Quality** Strong 86% (19/22)		**Exclusion:** None reported	**Parent report** NA	Mean changes observed across SIBS, SUD and TRF measures
				**Definition:** Internalising problems, withdrawal behaviours	**Teacher report** ● Direct behaviour Rating Single-Item Scale ● SIBS ● TRF; Internalising Scale+	Three participants no longer met established risk score
					**Clinician rating** NA	Evidence of functional relationship between intervention and internalising behaviours for all participants
					**Observations** NA	Increase in participation ratings for all participants
**Resilient Peer Treatment** Positive play skills, routine	Fantuzzo, Manz [36], USA	**NHMRC Level** III-1	**Total Sample:** *N* = 82 *Age*: 4.35 ± 0.47 *Gender*: 50% *M*, 50% *F* *Diagnosis*: Not reported	**Inclusion:** most socially withdrawn children across classrooms	**Self report** NA	Significant main effect for treatment for children in intervention group for collaborative play
		**Quality** Strong 93% (26/28)	**Intervention:** *N =* 38 *Age*: Not reported *Gende*r: Not reported *Diagnosis*: Not reported	**Exclusion:** None reported	**Parent report** NA	Significant main effect for treatment for intervention group for solitary play; intervention group showed less solitary play
			**Control:** *N* = 44 *Age*: Not reported *Gender*: Not reported *Diagnosis*: Not reported	**Definition:** socially withdrawn	**Teacher report** ● Penn Interactive Peer Play Scale ● Social Skills Rating System	No significant effects for associative or social attention play
					**Clinician rating** NA	Higher levels of interaction play for intervention group compared to control
					**Observations** ● Interactive Peer Pay Observational Coding System+	Intervention group rated significantly higher than control on play interaction and significantly lower on play disruption teacher rating scales
						Intervention group rated significantly higher than control on self-control and interpersonal skills on teacher rating scales
						Intervention group displayed lower levels of internalising, externalising and behaviour problems than control
**Social Effectiveness Therapy for Adolescents- Spanish version (SET-Asv)** Social skills, anxiety, fear, interpersonal functioning, participation in social activities	Garcia-Lopez, Olivares [37], Not reported	**NHMRC Level** III-2	**Total Sample:** *N = 25* *Age*: 20.83±0.79 *Gender*: 7*M*, 17 *F* *Diagnosis* (%): social phobia (100), avoidant personality (N.R.), selective mutism (10)	**Inclusion:** Generalised social anxiety	**Self report** ● SPAI; Social Phobia scale and Agoraphobia scale+ ● SAS-A; New Social Situations scale and Generalised Social Inhibition scale	Improvement between pre and post-test, maintained at 1and 5-year follow-up
**Cognitive-Behavioural Group Therapy for Adolescents (CBGT-A)** Social skills, problem-solving, cognitive restructuring		**Quality** Strong 82% (18/22)	**CBGT-A:** *N =* 8 *Age*: Not reported *Gender*: Not reported *Diagnosis* (%): social phobia (100)	**Exclusion:** None reported	**Parent report** NA	Social anxiety symptoms evident at 5-year follow-up, despite improvements
**Therapy for Adolescents with Generalised Social Phobia (IAFS)** Social skills, public speaking, initiate/maintain conversations			**SET-Asv**: *N* = 7 *Age*: Not reported *Gender*: Not reported *Diagnosis* (%): social phobia (100)	**Definition:** Social phobia, social anxiety disorder	**Teacher report** NA	At 5-year follow-up, SET-Asv and IAFS groups obtained lowest scores on all anxiety measures
			**IAFS:** *N* = 8 *Age*: Not reported *Gender*: Not reported *Diagnosis*: social phobia (100)		**Clinician rating** ● ADIS-C; Social Phobia Section	No significant differences between interventions in social anxiety scores at 5-year follow-up
					**Observations** NA	High effect sizes for all interventions
						43% of SET-Asv group no longer met DSM-IV criteria for social phobia at any follow-up period; 29% relapsed at 5-year follow-up
						12.5% of CBGT-A group no longer met DSM-IV criteria for social phobia at any follow-up period; 17.5% relapsed at 5-year follow-up
						25% of IAFS group no longer met DSM-IV criteria for social phobia at any follow-up period; 50% relapsed at 5-year follow-up
**Buddy Bench** Social involvement	Griffin, Caldarella [28], USA	**NHMRC Level** III-2	**Total Sample:** *N* = 388 *Age*: Grades 1 to 6 *Gender*: Not reported *Diagnosis*: Typically developing	**Inclusion:** Any child between Grades 1 to 6 at particular elementary school is Utah, USA	**Self report** NA	Students in 1^st^ to 3^rd^ grade playground extended 130 invitations to students on the bench 76 (58%) were accepted and led to play activities
		**Quality** Strong 86% (19/22)	**Teachers:** *N* = 21 *Age*: Not reported *Gender*: 1 *M*, 20 *F* *Diagnosis*: Typically developing	**Exclusion:** Kindergarten children at same school	**Parent report** NA	Average 1.03 students using the bench at any given time
				**Definition: S**olitary behaviour, not being engaged with other students or engaging in behaviour alone with no other students within five feet	**Teacher report** ● Treatment fidelity ratings; Reported they had taught students to use buddy bench, school-wide announcements, posted rules in classroom	Students on 4^th^ to 6^th^ grade playground extended 75 invitations to students using the bench 47 (63%) were accepted and led to play activities
					**Clinician rating** NA	Average 0.8 students using the bench at any given time
					**Observations** ● Number of students using bench ● Number of play invitations extended to students using bench ● Number of play invitations accepted by students using bench ● Successful teach-directed prompts to use bench ● Number of students engaged in solitary behaviour+	24% reduction in solitary behaviour from baseline for 1^st^ to 3^rd^ grade playground, statistically significant
						19% reduction in solitary behaviour from baseline for 4^th^ to 6^th^ grade playground, statistically significant
						When bench removed, solitary behaviour gradually returned to near baseline (13% increase from intervention phase)
						When bench re-introduced, solitary behaviour immediately decreased to near intervention levels (13% decrease)
**The Coping Bear Program** Relaxation techniques, cognitive restructuring	Hum, Manassis [38], Canada	**NHMRC Level** III-2	**Total Sample:** *N* = 88 *Age*: Not reported *Gender*: Not reported *Diagnosis*: Not reported	**Inclusion for clinical group:** rated within clinical range on Child Behaviour Checklist Internalising scale; attended more than 75% of therapy sessions; returned to the lab for post-treatment assessment	**Self report** ● MASC ● STAIC-S	Significant pre-post differences in CBL between comparison, improver and non-improver groups
		**Quality** Strong 95% (21/22)	**Clinical group:** *N* = 47 *Age*: Not reported *Gender*: Not reported *Diagnosis*: generalised anxiety, social anxiety or separation disorder	**Inclusion for control group:** rating within normal range on Child Behaviour Checklist internalising scale	**Parent report** ● CBCL; Internalising scale+	At post-test, improver and non-improver groups differ significantly in CBL scores
			**Control:** *N* = 41 *Age*: Not reported *Gender*: Not reported *Diagnosis*: Typically developing	**Exclusion:** None reported	**Teacher report** NA	Significant decrease in CBL scores pre-post for improver group
			**Treatment Improvers:** *N =* 11 *Age*: 10.58±1.19 *Gender*: 3*M*, 8 *F* *Diagnosis* (N): GAD (8), GAD and SOC (2), ADHD (2)	**Definition:** anxiety disorder, anxiety behaviour	**Clinician rating** NA	At both pre and post-test, comparison group differed from improvers and non-improvers on MASC scores
			**Treatment Non-improvers:** *N =* 13 *Age*: 10.46±1.29 *Gender*: 5 *M*, 8 *F* *Diagnosis* (N): generalised anxiety only (5), SOC only (2), separation anxiety only (1), SOC and separation anxiety (1), generalised anxiety and SOC (2), generalised anxiety and separation anxiety (1), generalised anxiety, SOC and separation anxiety (1)		**Observations** NA	At post-test, comparison group differed significantly from improvers on STAIC-S scores
					**EEG Task** ● Go/No Go tasks; Posterior P1 and frontal N2 components evaluated for correct No-go trials	No significant differences between groups of Go/NO Go accuracy, response duration, time allotment, Go response times and error No-go response times
						Greater P1 amplitudes for non-improvers compared to improvers or comparison
						Significant increase in N2 amplitude for improvers; decrease for non-improvers
**Cool Kids Program- For Parents** Psychoeducation, management strategies, cognitive restructuring, coping	Kennedy, Rapee [39], Australia	**NHMRC Level** III-2	**Total Sample:** *N = 71* *Age*: 47.07 ± 7.05 months *Gender*: Not reported *Diagnosis* (N): social phobia (70), generalised anxiety (1), specific phobia (37), separation anxiety (27), OCD (5), selective mutism (3), ODD (6), ADHD (3)	**Inclusion:** High score on laboratory measure of behavioural inhibition, one parent who met criteria for DSM-IV diagnosis of anxiety disorder	**Parent self-report** ● Depression Anxiety Stress Scale	Significant Time x Group interaction for BIQ inhibition, both maternal and paternal rating
		**Quality** Good 64% (18/28)	**Intervention:** *N* = 35 *Age*: 48.4±7.1 months *Gender*: 42% *M*, 58% *F* *Diagnosis*: Not reported	**Exclusion:** None reported	**Mother report** ● STSC; Approach subscale	Significant Time x Group interaction for Behaviour Inhibition Composite
			**Waitlist Control:** *N =* 36 *Age*: 45.8±6.9 months *Gender*: 49% *M*, 51% *F* *Diagnosis*: Not reported	**Definition:** Behavioural inhibition	**Parent report** ● BIQ ● PAS ● Child Anxiety Life Interference Scale-Preschool Version	Significant reduction in Global Inhibition, with significant Time x Group interaction
					**Teacher report** NA	46.7% of children in intervention group no longer had anxiety disorder, compared to 6.7% of control, significant difference
					**Clinician rating of parent** ● ADIS-C; Parent Version	Significant reduction in clinical severity ratings, Group x Time interaction
					**Observations** ● Behavioural inhibition across a number of activities with unfamiliar female assessor; Inhibition composite and Global Inhibition rating+	Significant main effect for time on maternal and paternal PAS-R ratings
						Significant Group x Time interaction for maternal and paternal ratings of life interference
						Maternal and paternal report of own anxiety did not show significant change over time or by group
**Cognitive bias modification training** Interpretation bias	Klein, Rapee [40], Australia	**NHMRC Level** III-2	**Total sample:** *N* = 83 *Age*: 9.2±1.5 *Gender*: 43 *M*, 40 *F* *Diagnosis* (%): generalised anxiety (89.2), social phobia (68.7), separation anxiety (44.6), other anxiety disorders (n = 55), mood disorder (n = 12), behaviour disorder (n = 17)	**Inclusion:** Primary anxiety disorder, aged 7–12 years.	**Self-report** ● Spence Children’s Anxiety Scale- Child Version	No main effects or interactions for social threat or general threat scenarios
		**Quality** Strong 82% (23/28)	**Positive training:** *N =* 40 *Age*: 9.1±1.6 *Gender*: 22 *M*, 18 *F* *Diagnosis*: Not reported	**Exclusion:** Life threatening suicidal ideation, in physically or sexually abusive environments, under current psychological treatment, significantly intellectually impaired, had unmanaged psychotic symptoms	**Parent report** ● Spence Children’s Anxiety Scale- Parent Version	Significant Time x Set interaction for non-threat scenarios; children had difference scores over time depending on the scenario set of interpretation task
			**Neutral training:** *N* = 43 *Age*: 9.4±1.4 *Gender*: 21 *M*, 22 *F* *Diagnosis*: Not reported	**Definition:** Clinically anxious, anxiety disorder	**Teacher report** NA	Significant reduction in interpretation biases for social threat scenarios in positive group No significant reduction for neutral group
					**Clinician rating** ● ADIS-C; Parents and child version	No significant effect of positive training on children’s self-reported social, generalised or separation anxiety
					**Performance** ● Interpretation task; Asked to read aloud 3 sets of 15 scenarios presented on a computer screen and choose the ending they thought would best fit; Non-threat, social threat and physical threat scenarios+	Significant reduction in social anxiety in mother and father-reports
**UTalk- Interpersonal Psychotherapy Adolescent Skills Training** Social anxiety, depression, peer relationships, approaching other peers, coping with peer victimisation	La Greca, Ehrenreich-May [41], USA	**NHMRC Level** IV	**Total sample:** *N* = 14 *Age*: 15.64±1.28 *Gender*: 21.4% *M*, 78.6% *F* *Diagnosis* (%): social anxiety (71)	**Inclusion:** Elevated levels of symptoms of social anxiety of depression, elevated levels of relational or reputational peer victimisation on screening measures	**Self report** ● Revised Peer Experiences Questionnaire ● SAS-A+ ● Center for Epidemiological Studies Depression Scale ● Youth Self Report; Aggression subscale ● Cyber-Peer Experiences ● Perceived Social Support Scale	Significant decrease from baseline to post-intervention for clinician ratings of severity of ADIS-C and CGI
		**Quality** Good 77% (17/22)		**Exclusion:** Aggressive behaviour, overt victimisation	**Parent report** NA	Significant decrease in relational and reputational peer victimisation
				**Definition:** Social anxiety	**Teacher report** NA	Significant decrease in report of cyber peer victimisation
					**Clinician rating** ● ADIS-C ● CGI ● Columbia-Suicide Severity Scale	Significant decrease in social anxiety and depression symptoms
					**Observations** NA	Increases in perceived social support from friends
**Second Life** Self-expression	Lee [42], South Korea	**NHMRC Level** III-3	**Total sample:** *N* = 60 *Age*: 5^th^ Grade *Gender*: 34 *M*, 26 *F* *Diagnosis*: Typically developing	**Inclusion:** 5^th^ grade elementary class in participating school; group membership determined by scores on shyness scale	**Self report** ● Revised Cheek and Buss Shyness and Sociability Scale+ ● Self-Administered Assertiveness scale	High shyness group had a lower baseline level of self-expression than low shyness group
		**Quality** Good 77% (17/22)	**High shyness:** *N* = 30 *Age*: Not reported *Gender*: 16 *M*, 14 *F* *Diagnosis*: Typically developing	**Exclusion:** None reported	**Parent report** NA	High shyness group showed an average increase in self-expression of 3.14
			**Low shyness:** *N* = 30 *Age*: Not reported *Gender*: 18 *M*, 12 *F* *Diagnosis*: Typically developing	**Definition:** Feeling of apprehension, discomfort of awkwardness in unfamiliar situations/with unfamiliar people	**Teacher report** NA	Low shyness group showed an average increase in self-expression of 1
					**Clinician rating** NA	High shyness group had significantly greater improvements, compared to low shyness group
					**Observations** NA	
**Social Skills Training Facilitated Play (SST-FP)**	Li, Coplan [43], China	**NHMRC Level** III-2	**Total sample:** *N* = 16 *Age*: 4.68±0.28 *Gender*: 8 *M*, 8 *F* *Diagnosis*: Typically developing	**Inclusion:** Aged 4–5 years, parent-rated shyness below top 25% of CBQ, nominated by teacher as top 5 shy children, no known developmental/psychiatric disorder	**Self report** NA	Main effect of Time for peer interaction during free play
Initiating/maintaining conversions, understanding/expressing feelings, emotion regulation, peer interaction		**Quality** Strong 96% (27/28)	**Intervention:** *N* = 8 *Age*: Not reported *Gender*: 4 *M*, 4 *F* *Diagnosis*: Typically developing	**Exclusion:** Known psychiatric or developmental disorder	**Parent report** ● CBQ	Intervention group engaged in significantly more peer interaction than control, immediately following intervention
			**Comparison:** *N* = 8 *Age*: Not reported *Gender*: 4 *M*, 4 *F* *Diagnosis*: Typically developing	**Definition:** Excessive wariness and unease in social novelty and perceived social evaluation	**Teacher report** NA	Difference maintained at 2-month follow-up
					**Clinician rating** NA	Main effect of Time for frequency of prosocial behaviours
					**Observations** ● Adapted Play Observation Scale; Time spent in peer interaction, frequency of prosocial behaviours+ ● Observation during self-presentation speech sessions; Amount of eye contact, nervous affect, positive body posture	Intervention group engaged in significantly more prosocial behaviours than control, immediately following intervention
						Difference maintained at 2 month follow-up
						Main effect of Time for speech performance
						Intervention group performed significantly better during speeches than control, immediately following intervention
**Implosive, Counselling and Conditioning Approach**	Lowenstein [44], England	**NHMRC Level** III-2	**Total sample:** *N* = 22 *Age*: 9–16 years *Gender*: 6 *M*, 16 *F* *Diagnosis*: Not reported	**Inclusion:** Known to teachers as timid, totally or virtually eschewed social contact, scores below 8 on MPI Extroversion scale	**Self report** NA	Children in intervention group showed significantly lower timidity ratings post-intervention, compared to control
Eye contact, interest in communication with others, mixing socially, assertiveness		**Quality** Good 64% (18/28)	**Intervention:** *N* = 11 *Age*:9–16 years *Gender*: Not reported *Diagnosis*: Not reported	**Exclusion:** Score above 5 on MPI Psychoticism scale	**Parent report** NA	Significant increase in extroversion for intervention group, compared to control
			**Control:** *N* = 11 *Age*:9–16 years *Gender*: Not reported *Diagnosis*: Not reported	**Definition:** Easily frightened, timid, bashful, shrinking from approach or familiarity	**Teacher report** ● MPI ● Timidity rating+	
					**Clinician rating** NA	
					**Observations** NA	
**Cool Little Kids**	Luke, Chan [45], Hong Kong	**NHMRC Level** III-2	**Total sample:** *N* **=** 57 *Age*: 3.91±0.60 *Gender*: 35 *M*, 22 *F* *Diagnosis*: Typically developing	**Inclusion:** Level of behavioural inhibition, attending a local kindergarten, no known childhood developmental disorder, not receiving services for learning disabilities	**Self report** NA	Significant main effect of Time on anxious shyness
Parental overprotection, avoidance		**Quality** Strong 86% (24/28)	**Intervention:** *N* = 25 *Age*: $\bar{x}$ 3.84 *Gender*: 11 *M*, 14 *F* *Diagnosis*: Typically developing	**Exclusion:** Known childhood developmental disorder, receiving services for learning disabilities	**Parent report** ● BIQ	Significant Time x Group interaction on anxious shyness
			**Control:** *N* = 20 *Age*: $\bar{x}$ 3.98 *Gender*: 16 *M*, 4 *F* *Diagnosis*: Typically developing	**Definition:** Behavioural inhibition	**Teacher report** ● BIQ ● Chinese Shyness Scale+ ● Social Competence Inventory ● CBS	Intervention group showed significant decrease in anxious shyness, compared to control
					**Clinician rating** NA	Significant main effect of Time on social initiative
					**Observations** NA	Significant main effect of Time on internalising problems
**Pyramid Program** Problem-solving, assertive communication, relaxation, emotional expression	McKenna, Cassidy [46], Northern Ireland	**NHMRC Level** III-2	**Total sample:** *N* = 82 *Age*: 7–8 years *Gender*: Not reported *Diagnosis*: Not reported	**Inclusion:** SDQ scores in normal range, displaying subtle changes in withdrawal, known to be experiencing difficulty at home OR scored in borderline or abnormal range for SDQ Emotional or Peer Problems, but no comorbid externalising problems	**Self report** NA	Changes in emotional symptoms and peer problems dependent on group membership
		**Quality** Strong 91% (20/22)	**Intervention:** *N* = 57 *Age*: 7–8 years *Gender*: 41.7% *M*, 48.3% *F* *Diagnosis*: Not reported	**Exclusion:** Those not meeting above criteria were included in control group	**Parent report** NA	No significant interaction for prosocial skills
			**Control:** *N* = 31 *Age*: 7–8 years *Gender*: 50.6% *M*, 49.4% *F* *Diagnosis*: Not reported	**Definition:** Behavioural withdrawal, wariness in the face of novelty and social evaluation	**Teacher rating** ● SDQ; Emotional, Peer Problems and Pro-social subscales+	33.3% of Intervention group in abnormal range for emotional symptoms at baseline; decreased to 6.3% post-intervention; increased to 10% at 12-week follow-up
					**Clinician rating** NA	22.8% of Intervention group in abnormal or borderline range for peer problems at baseline; decreased to 3.2 post-intervention; increased to 5.8% at 12-week follow-up
					**Observations** NA	35.6% of Intervention group experiencing peer exclusion at baseline; decreased to 13.7% post-intervention; increased to 24.3% at 12-week follow-up
**INSIGHTS** Academic development, critical thinking, math, language, empathy, problem solving	O’Connor, Cappella [47], USA	**NHMRC Level** III-2	**Total sample:** *N* = 345 *Age*: 5.38±0.61 *Gender*: 50% *M*, 50% *F* *Diagnosis*: Typically developing	**Inclusion:** Enrolled in kindergarten at participating school, first 10 to sign up	**Self report** NA	No significant main effect for treatment
		**Quality** Strong 86% (24/28)	**Intervention:** *N* = 183 *Age*: Not reported *Gender*: Not reported *Diagnosis*: Typically developing	**Exclusion:** None reported	**Parent report** ● School-Aged Temperament Inventory	Children with shyer temperaments showed lower scores on critical thinking, language and math
			**Control:** *N* = 162 *Age*: Not reported *Gender*: Not reported *Diagnosis*: Typically developing	**Definition:** fearful, anxious, wary, and reluctant to take part in interactions with others in situations that involve novelty or actual/perceived judgement	**Teacher report** ● Academic Competence Evaluation Scale; Critical thinking, reading/writing, mathematics subscales	Significant Treatment x Time x Shy effect for critical thinking and math
					**Clinician rating** NA	Shy children in treatment group experienced stable math skills, compared to a decrease in control group
					**Observations** ● Behavioural Observation of Students in Schools; Frequency of engagement in academic activities+	Shy children in treatment group increased critical thinking skills, compared to decrease in control group
						Improvement in behavioural engagement partially mediated relationship between treatment and critical thinking, and math
**Parent education program** Child temperament	Rapee and Jacobs [48], Australia	**NHMRC Level** IV	**Total sample:** *N* = 7 *Age*: 56.3±4.1 months *Gender*: 7 *M* *Diagnosis*: Typically developing	**Inclusion:** Top 25% on Childhood Temperament Questionnaire-Approach scale	**Self report** NA	Significant effect of Time on CTQ across pre-, post-intervention and 6-month follow-up
		**Quality** Strong 85% (17/20)		**Exclusion:** Already receiving therapy	**Parent report** ● Childhood Temperament Questionnaire- Australian Adaptation ● Revised Children’s Manifest Anxiety Scale-Modified +	Change from pre- to post-intervention not significant
				**Definition:** socially withdrawn	**Teacher report** NA	Change from pre-intervention to follow-up significant
					**Clinician rating** NA	Significant effect of Time on anxiety across pre-, post-intervention and 6 month follow-up
					**Observation** NA	Significant changes pre- to post-intervention, and pre-intervention to follow-up
	Rapee, Kennedy [49], Australia	**NHMRC Level** III-2	**Total sample:** *N* = 146 *Age*: $\bar{x}$ 46.8 months *Gender*: Not reported *Diagnosis*: Typically developing	**Inclusion:** Score above 30 on STSC Approach scale, above cut-off on 3 behaviours on behavioural observation	**Self report** NA	Significant reduction in anxiety disorders at 12-month follow-up for Intervention group
		**Quality** Good 75% (21/28)	**Intervention:** *N* = 73 *Age*: 47.3±5.1 months *Gender*: 40% *M*, 60% *F* *Diagnosis*: Typically developing	**Exclusion:** None reported	**Parent report** ● STSC ● Temperament Assessment Battery for Children-Revised ● ADIS-C; Parent Version+	Inhibition at 12 months was not influenced by group membership
			**Control:** *N* = 73 *Age*: 46.1±4.4 months *Gender*: 51% *M*, 49% *F* *Diagnosis*: Typically developing	**Definition:** Inhibited or withdrawn temperament	**Teacher report** NA	
					**Clinician rating** NA	
					**Observations** ● Behavioural inhibition: Total amount talking, total time near mother, duration of staring at peers, frequency of approach to strangers and peers	
**Cognitive-behavioural approach-based social skills training** Internalising behaviours	Sang and Tan [50], China	**NHMRC Level** III-2	**Total sample:** *N* = 29 *Age*: 9–12 *Gender*: Not reported *Diagnosis*: Typically developing	**Inclusion:** Suspected of internalising disorder, aged between 9 and 12, speaking Chinese, basic reading/writing skills	**Self report** NA	Significant decrease in anxiety for Intervention group at post-intervention and 2 month follow-up
		**Quality** Good 71% (20/28)	**Intervention:** *N* = 16 *Age*: 9–12 *Gender*: Not reported *Diagnosis*: Typically developing	**Exclusion:** None reported	**Parent report** ● CBCL Internalising scale + ● Social Competence Scale	Significant increase in anxiety for Control at post intervention and 2 month follow-up
			**Control:** *N* = 13 *Age*: 9–12 *Gender*: Not reported *Diagnosis*: Typically developing	**Definition:** Internalising disorder	**Teacher report** NA	Significant decrease in depression for Intervention group at post-intervention and 2 month follow-up
					**Clinician rating** NA	Significant increase in depression for Control at post intervention and 2 month follow-up
					**Observations** NA	Significant decrease in withdrawal for Intervention group at post-intervention and 2 month follow-up
						Significant increase in withdrawal for Control at post intervention and 2 month follow-up
**Group cognitive behavioural therapy** Relaxation, social skills, overall shyness	Umeh [51], Lagos	**NHMRC Level** III-2	**Total sample:** *N* = 36 *Age*: 14.63±2.47 *Gender*: Not reported *Diagnosis*: Typically developing	**Inclusion:** highest 36 scores on SS-34	**Self-report** ● Shyness Scale 34+	Significant effect of between-subject factor groups
		**Quality** Good 79% (22/28)	**Intervention:** *N* = 18 *Age*: 10–19 *Gender*: Not reported *Diagnosis*: Typically developing	**Exclusion:** None reported	**Parent report** NA	59% of overall variance accounted for by treatment
			**Control:** *N* = 18 *Age*: 10–19 *Gender*: Not reported *Diagnosis*: Typically developing	**Definition:** Discomfort in social situations	**Teacher report** NA	Intervention group showed reduction in shyness levels, compared to Control
					**Clinician rating** NA	
					**Observations** NA	
**Emotion recognition training program** Emotion recognition, perception of happiness in others	Rawdon, Murphy [52], UK/Ireland	**NHMRC Level** II	**Total sample:** *N* = 92 *Age*: 15.77±0.66 *Gender*: 33 *M*, 59 *F* *Diagnosis*: Typically developing	**Inclusion:** Score above 21 on SPAIC-C	**Self-report** ● SPAI-C+ ● BFNE-R ● SCARED ● RCADS-MDD ● Emotion recognition balance point	Significant main effect of Time of SPAI-C total score
		**Quality** Strong 96% (27/28)	**Intervention:** *N* = 49 *Age*:15.71±0.68 *Gender*: 17 *M*, 32 *F* *Diagnosis*: Typically developing	**Exclusion:** Score below 21 on SPAI-C; parent reported diagnosed mental health disorder and/or attending mental health professional	**Parent report** NA	Significant decrease in SPAI-C scores from pre-intervention to 2-week follow-up
			**Control:** *N =* 43 *Age*:15.84±0.65 *Gender*: 16 *M*, 27 *F* *Diagnosis*: Typically developing	**Definition:** Social anxiety	**Teacher report** NA	Significant decrease in SPAI-C scores from post-intervention to follow-up
					**Clinician rating** NA	No different in SPAI-C scores from pre- to post-intervention
					**Observations** NA	No main effect of Training or Time x Training interaction
						Time x Training interaction of balance point scores; significant effect of Time on intervention group, but not control group, for balance point scores
						Main effect of Time of SCARED total scores
						Time x Training interaction on RCADS-MDD; significant effect of Time on intervention group but not control

Notes

^1^ NHRMC hierarchy: Level 1 Systematic reviews; Level II Randomized control trials; Level III–1 Pseudo-randomized control trials; Level III–2 Comparative studies with concurrent controls and allocation not randomized (cohort studies), case control studies, or interrupted time series with a control group; Level III–3 Comparative studies with historical control, 2 or more single-arm studies, or interrupted time series without a control group; Level IV Case series.

^2^ Methodological quality: Strong > 80%; good 60–79%; adequate 50–59%; poor < 50%.

ADHD = Attention Deficit/Hyperactivity Disorder; BFNE-R = Brief Fear of Negative Evaluation- Revised; BIQ = Behavioural Inhibition Questionnaire; CBCL = Child Behaviour Checklist; CBS = Child Behaviour Scale; CGI = Clinical Global Impressions; CIRP = Children’s Intervention Rating Profile; DSM-IV = Diagnostic and Statistical Manual of Mental Disorders; GAD = Generalised Anxiety Disorder; K-GAS = Children’s Global Assessment Scale; MASC = Multidimensional Anxiety Scale for Children; MPI = Maudsley Personality Inventory; NOS = Not Otherwise Specified; OCD = Obsessive Compulsive Disorder; ODD = Oppositional Defiance Disorder; PAS = Preschool Anxiety Scale; RCADS-MDD = Revised Child Anxiety and Depression Scale–Major Depressive Disorder; SAS = School Anxiety Scale; SAS-A = Social Anxiety Scale for Adolescents; SCARED = Screen for Child Anxiety Related Emotional Disorders; SDQ = Strengths and Difficulties Questionnaire; SIBS = Student Internalising Behaviour Screening; SOC = Sense of coherence; SPAI = Social Phobia and Anxiety Inventory; SPAI-C = Social Phobia and Anxiety Inventory for Children; STAI-C = State-Trait Anxiety Inventory for Children; STSC = Short Temperament Scale for Children; SUD = Subjective units of distress; TRF = Teacher Report Form; + main shyness outcome measure extracted for meta-analysis.

### Data items, risk of bias and synthesis of results

Risk of bias in the included studies was assessed at an individual study level using the Kmet appraisal checklist [26]. Risk of bias was minimised in this process by having a full overlap between independent abstract and article reviewers, and by two independent assessors independently scoring 100% of the methodological quality of included studies. Final study selection and quality assessment were the result of consensus-based ratings. Discrepancies were resolved by involving a third reviewer. No author of this review was affiliated with any of the included studies. Extracted data were synthesised in relation to the methodological characteristics of each included study and the findings of individual studies with regards to the treatment outcomes of shyness interventions.

### Meta-analysis

Using the extracted data from the main outcome measure related to shyness, estimates were calculated of pooled effect sizes weighted by sample size using random-effects models for summary statistics. To determine potentially confounding variables, effect sizes of shyness interventions were grouped by setting (school, clinic and/or home), focus (child and/or parents), mode of delivery (individual and/or group sessions), and rater of outcome measures (child, parents, clinician and/or teacher). The Hedges-*g* formula for standardized mean difference (SMD) with a 95% confidence interval (95% CI) was used to report effect sizes. A test for overall effect for each intervention setting, mode, focus and outcome rater produced a weighted effect size (*z*). Tests for heterogeneity were conducted to identify inconsistency in treatment effects, included *I*^2^ and chi-square (*Q*). All statistical analyses were performed using software package Comprehensive Meta-Analysis Version 3.3.070 (Biostat; Englewood, NJ, USA).

Within-groups effects were examined by analysing the pre-post data for studies both with and without control groups. The benefit of within-groups analyses is that it allows the examination of the effect of an intervention in and of itself, without controls. Between-groups analyses (comparing results of control group to that of intervention group) were also conducted. This allows comparison of different forms of interventions against each other.

## Results

### Systematic review

#### Study selection

**Fig 1:**
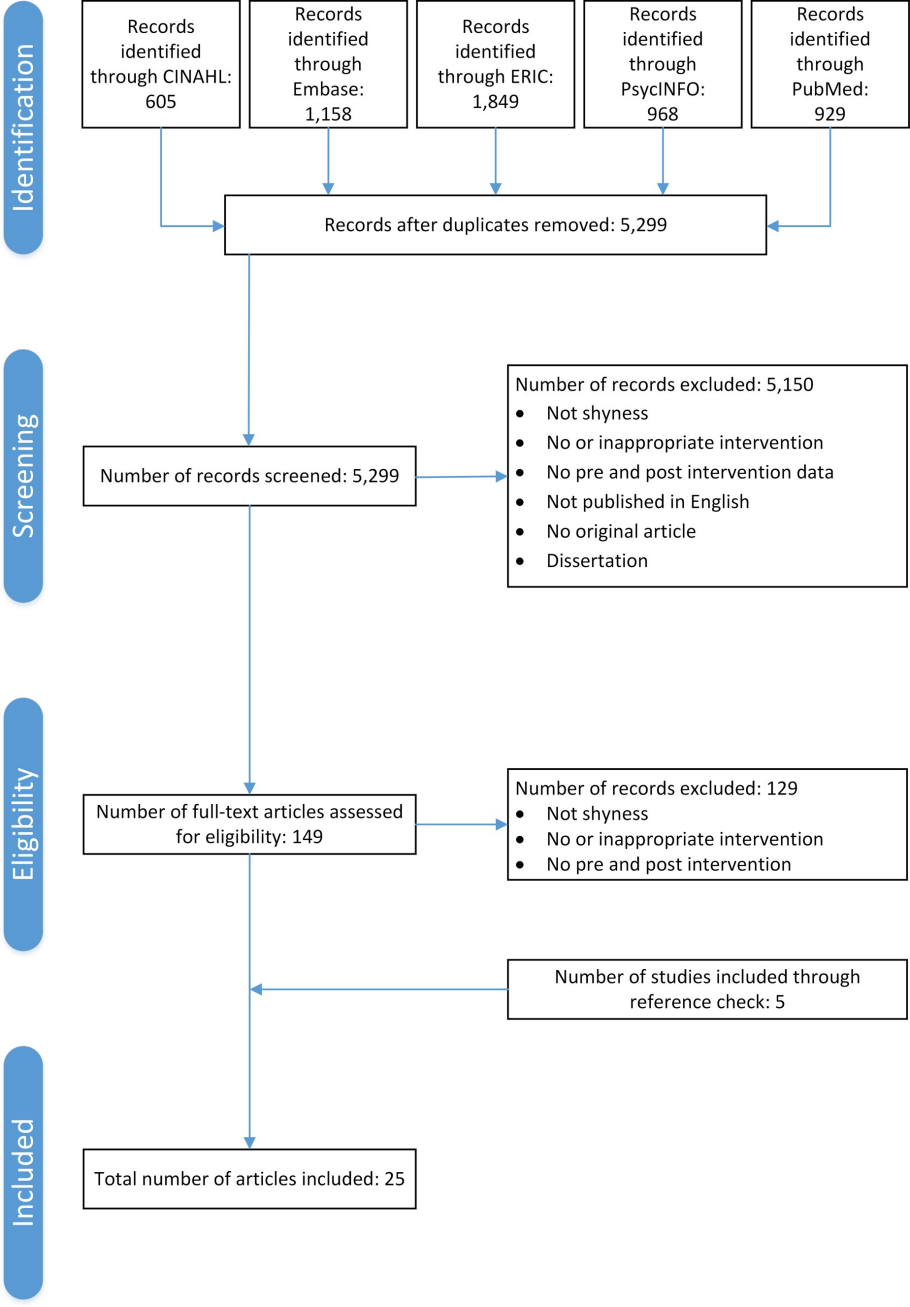
Flow diagram of the review process according to Preferred Reporting Items for Systematic Reviews and Meta-Analyses (PRISMA). Adapted from Moher et al.

A total of 4,864 articles were identified (CINAHL: n = 605, Embase: n = 1158, ERIC: n = 1849, PsycINFO: *n* = 968 and PubMed: n = 929). After the removal of duplicate articles, 5299 abstracts were screened. A total of 149 studies were assessed at a full text level for eligibility. Of these, 129 were excluded and 20 were included (see Fig 1). No studies were excluded due to poor quality. An additional five studies were included through searching the reference lists of the 20 studies that met the inclusion criteria. This resulted in a total of 25 included studies.

#### Participants of studies included in the systematic review

The total number of participants across the 25 included studies was 1,895, with the average participants across studies 75.8. Griffin, Caldarella [28] had the largest sample of 388 participants and Cook, Xie [29] the smallest sample of 5 participants. The average age of total participants across the studies was 9.1 years (SD = 5.4), with the average age of the total sample not reported in nine studies. Of the 25 studies, only five had more male than female participants, with four studies not reporting the gender of the total or sub-samples. While a range of diagnoses were reported across some studies, 13 studies reported the sample to be typically-developing and five studies did not report diagnosis. Studies were conducted across nine countries, with the highest number conducted in the USA (*n* = 10), followed by Australia (*n* = 4). Additional details on participant characteristics are reported in Table 2.

#### Study design, methodological quality and risk of bias of studies included in the systematic review

Most studies were randomised or pseudo-randomised control trials, with only three employing a multiple baseline design (see Table 2). The methodological quality for each study according to Kmet criteria is reported in Table 2. The average methodological quality rating across all studies was 83.4% (SD: ±8.7, range: 64–96%), indicating “strong” methodological quality. Of the studies, 17 were rated as “strong”, with all others rated to have “good” methodological quality. No study was rated to have adequate or poor methodological quality.

#### Shyness outcome measures

While studies reported several outcome measures, only those relevant to shyness and/or social anxiety were the focus of this review. Across categories of self-report, parent-report, teacher-report, clinician-rating and observation measures of shyness, self- reported (*n* = 13) and parent-reported (*n* = 13) shyness outcome measures were most frequently used and clinician-rating was used least across studies (*n* = 7; see Table 2). Using the categories of outcome measures above, nine studies used two different types of outcome measures, seven studies used only one type of outcome measure, and nine studies used three or more types out outcome measures.

#### Interventions

The majority of studies included an intervention that was delivered weekly (*n* = 15), in a child group format (*n* = 14), in the school setting (*n* = 10). Only four studies reported session durations of 40 minutes, with 14 reporting sessions for 60 minutes or longer. Intervention delivery was reported to be at least 7 weeks in 17 studies (see Table 3).

**Table 3:** Characteristics of interventions.

Intervention/Target Skills	Procedure	Interventionists	Duration/Setting/Mode of Delivery	Tailoring/Modifications	Active Ingredients
**Social Effectiveness Training for Children (SET-C)** Social anxiety and fear, social skill, interpersonal functioning, participation in social activities	Stand-alone educational session was held for parents and children about social phobia. Treatment sessions consisted of 4–6 children with a social skills training component. One social skill was taught each week using instruction, modelling, rehearsal and corrective feedback. Followed by 90 min of peer-generalisation with non-anxious peers. Different peers were used each week. Children were assigned homework on each week’s content. Once a week, in vivo exposure sessions were conducted of anxiety-inducing scenarios until anxiety dissipates (45–75 min).	*Therapist* Qualifications: Not reported Relationship: Not reported	**Dosage** 36 hours *Individual sessions*: 1 x 60–90 minutes weekly sessions, for 12 weeks *Group sessions*: 1 x 60–90 minutes weekly sessions, for 12 weeks	None reported	● Psychoeducation ● Social skills training ● Therapist modelling ● In vivo exposure ● Behaviour modification
Beidel, Turner [30], Beidel, Turner [31]			**Mode** Child individual Child group		
			**Setting** Not reported		
**Problem-solving and conversational skills training** Recognising a problem, defining a problem, generating solutions, evaluating consequences, determining best solution, implementing a solution, listening, talking about oneself, initiating conversations, making requests of others	Four problem-solving skills training sessions. In the first session, therapists provide a rationale for learning skills, remaining sessions involve applying skills to interpersonal problems. Students complete a worksheet and discuss in subsequent session. Social skills training is conducted for next four sessions. Each session focused on a different topic, discussed and modelled by therapist and rehearsed by participants. Participants role-play skills with one another and are given feedback by therapists. Participants are assigned homework to practice skills at home.	*Therapist* Qualifications: Clinical psychology interns Relationship: Not reported	**Dosage** 7 hours *Group sessions*: *1* x 40-minute group sessions weekly, for 8 weeks (4 x problem solving skills sessions, 4 x conversational skills training sessions)	None reported	● Therapist modelling ● Social skills training
Christoff, Scott [32]			**Mode** Child group		
			**Setting** School		
**Turtle Program** Social skill, introducing self, eye contact, communication, relaxation, expressing emotions, working together, exposure to fear	**Parent component:** Parents attended 8 sessions. Sessions included psychoeducation and teaching and coaching of each Child-Direction Interaction, Bravery-Directed Interaction and Parent-Direction Interaction. Parents learn to adopt a “step behind” approach, provide praise for their child’s behaviours, apply skills in anxiety-provoking social situations for their child, and distinguish between anxiety and oppositional behaviours. Coaching sessions involved dyadic parent-child coaching. Parents received instructions for out of session exposures. The final session was a “graduation party” were parents were coached to use their skills	*Therapist* Qualifications: Not reported Relationship: Not reported	**Dosage** *Parents*: 12 hours *Parent sessions*: 1 x 90-minute sessions weekly, for 8 weeks *Child*: 12 hours *Child group sessions*: 1 x 90-minute sessions weekly, for 8 weeks	None reported	● Social skills training ● Psychoeducation ● Therapist modelling ● Behaviour modification ● In vivo exposure
Chronis-Tuscano, Rubin [33]	**Child component:** Adapted from Social Skills Facilitated Play. Children attended 8 group sessions. Session topics included learning to introduce yourself, making eye contact, relaxation, communicating to keep friends, facing your fears, expression emotions, working together and group activities. Skills were taught using puppets and games. After teaching portion, children engaged in free play and group activities, using modelling, guided participation and reinforcement of social skills by therapists. Activities, such as Show and Tell, were incorporated to allow for exposure to feared situations.	*Parent* Qualifications: NA Relationship: Parent of participating child	**Mode** Child group Parent group		
			**Setting** Clinic		
**The Courage and Confidence Mentor Program** Internalising problems	Mentors were any educational professional at the student’s school that participated in a 60 min training session. Prior to intervention, mentors held 2 x 40 min sessions. The first session was to build rapport and present *life bus metaphor*, used to normalise emotion and provide language to talk about emotions. The second session comprised a brief review of content and “courage tools”. The intervention consisted 0f a) assignment of a mentor with unconditional positive regard; b) morning meetings for positive interaction, words of encouragement and pre-correction of problems; c) daily mentoring of performance and d) afternoon meetings for positive interaction and performance-based feedback. During meetings, students would provide daily ratings of distress.	*Mentor*: School psychologist and special education resource teacher; 60-min show and tell training, provided materials to support implementation	**Dosage** 6 hours *Individual content sessions*:2 x 40 min content sessions at beginning of intervention *Individual mentor sessions*: 2 x 5–10 minute sessions daily, for 3 weeks	Altering nature of communication between mentor and student	
Cook, Xie [29], Fiat, Cook [34]			**Mode** Child individual	For older students, used toy bus and figures to represent students themselves, to make metaphor more concrete	
			**Setting** School		
**Play Skills for Shy Children** Social skills, initiating and maintaining interactions, expressing and understand emotions, relaxation techniques	Each session involved a 5-minute free play period, followed by circle time sessions to provide didactic content. Leaders focused on specific set of skills each week, using songs, games and puppets to teach content. The first three sessions focused on initiating and maintaining peer interactions. The next three focused on understanding and expressing feelings. Sessions then comprised of leader-facilitated free play, where leaders prompted, modelled and reinforced skills discussed in circle time. Each session ended with a structured positive social activity.	*Therapist* Qualifications: Female leaders with previous early childhood education experience, trained by senior authors Relationship: Not reported	**Dosage** 8 hours *Group sessions*: 1 x 1-hour sessions a week for 7 weeks *Booster session*: 1x 1-hour booster session, approx. 1 month after completion	None reported	● Behaviour modification
Coplan, Schneider [35]			**Mode** Child group		
			**Setting** Community centre		
**Resilient Peer Treatment** Positive play skills, routine Fantuzzo, Manz [36]	Play Supporter arranges play corner, a designated area for Play Partner (participant) and Play Buddy to use for play. Play Supporter then spends a few minutes one-on-one with Play Buddy identify what behaviours resulted in positive interactions with Partner. During play sessions, Supporters observes session and makes supportive comments to Partner and Buddy about their interactive play at end of session.	*Parent* Play Supporters Qualifications: NA Relationship: Family volunteers with high levels of supportive and nurturing actions with children *Peer* Play Buddy Qualifications: NA Relationship: School peers with highest levels of prosocial peer play interactions	**Dosage** Total hours not reported *One on one play sessions*: 15 x play sessions; 3 sessions per week for 5 weeks	None reported	● Therapist modelling ● Behaviour modification ● Social skills training ● Peer mediation ● Behavioural modification
			**Mode** Child group		
			**Setting** School		
			**Mode** Child group		
**Social Effectiveness Therapy for Adolescents- Spanish version (SET-Asv)**	Participants first attend a group education session. Social skills training and exposure components are conducted during the first 13 weeks. Social skills training sessions involve 60-minute group sessions, learning to maintain conversation, give and receive compliments, establish and maintain friendships. Exposure sessions are conducted concurrently, in an individual format. Programmed practice sessions are completed once social skills training and exposure sessions are finished, aiming to maximise generalisation to natural environment	*Therapist* Qualifications: Not reported Relationship: Not reported	**Dosage:** 24.5 hours *Individual group sessions*: once off session, 60 minutes *Group sessions*: 1 x 60 min group sessions a week for 13 weeks *Individual exposure sessions*: 1 x 30 min individual sessions a week *Individual programmed practice*: 4 x 60 min sessions	Spanish language Adapted for adults	● Psychoeducation ● Social skills training ● In vivo exposure
Garcia-Lopez, Olivares [37]			**Mode** Child group Child individual		
			**Setting** Not reported		
**Cognitive-Behavioural Group Therapy for Adolescents (CBGT-A)**	Involves two phases with eight sessions each: 1) Educative and skills building and 2) Exposure. In first phase, therapist provides information about the program and delivers presentation on social phobia. The skills building unit involves teaching social skills, problem solving and cognitive restructuring. The second phase involves behaviour rehearsals and in vivo exposures within the session and as homework.	*Therapist* Qualifications: Not reported Relationship: Not reported	**Dosage** 24 hours *Group sessions*: 16 x 90-minute group treatment sessions over 14 weeks. First 4 sessions conducted within 2 weeks; remaining sessions happen weekly	None reported	● Psychoeducation ● Social skills training ● In vivo exposure ● Cognitive restructuring
Garcia-Lopez, Olivares [37]			**Mode** Child group		
			**Setting** Not reported		
**Therapy for Adolescents with Generalised Social Phobia (IAFS)**	Sessions included social skills training, exposure and cognitive restructuring techniques. Exposure to social stations used peer assistants to initiate/maintain conversations or public speaking in front of group mates and therapist for 5–10 mins. Exposure tasks are recorded and used as feedback. The last session focuses on relapse prevention. Weekly individual counselling sessions scheduled as needed.	*Therapist* Qualifications: Not reported Relationship: Not reported *Peer* Qualifications: NA Relationship: Unknown to the participant	**Dosage** 18 hours *Group sessions*: 1 x 90 min group sessions, for 12 weeks *Optional individual sessions*: weekly, as needed	Optional individual counselling or telephone consultation with therapist	● Social skills training In vivo exposure ● Cognitive restructuring ● Video modelling
Garcia-Lopez, Olivares [37]			**Mode** Child group Child individual		
			**Setting** Not reported		
**Buddy Bench** Social involvement	Observers, teacher and principal were trained in the intervention by principal investigator. Two specially decorated benches are placed in two playgrounds in the school. Teachers instructed students on how to use the bench, posted rules in every classroom and issues a daily school-wide announcement reminding students the use the bench. Students were instructed, if they felt alone, to sit at the bench. If someone invites them to play, say “yes” or “no thank-you”. If they saw a student at the bench, students were instructed to invite them to play.	*Teacher*: Qualifications: 21 years experience as educator Relationships: Teachers of participant students *Peer* Qualifications: NA Relationship: Grades 1 to 6 classmates	**Dosage** 10 weeks *School-wide intervention*: benches were placed in playgrounds for 10 weeks **Mode** School-wide	None reported.	● Peer mediation
Griffin, Caldarella [28]		*Therapist* Qualifications: Not reported Relationship: Not reported	**Setting** School		
**The Coping Bear Program** Relaxation techniques, cognitive restructuring	Participants take part of group cognitive behavioural therapy program. Participants are taught to recognise anxious physiological symptoms, maladaptive cognitions and attributional thinking. Participants learn helpful attitudes, positive self-talk and relaxation techniques to alleviate anxiety and modify problematic strategies. Exposure activities are then used to prompt participants to use newly learned strategies. A concurrent parent group program teaches parents to support children’s new use of strategies	*Therapist* Qualifications: Not reported Relationship: Not reported	**Dosage** *Children*: total hours not reported *Group sessions*: 12-week program *Parents*: Total hours not reported *Parent group program*: 12-week program	None reported.	● Cognitive restructuring ● In vivo exposure ● Psychoeducation
Hum, Manassis [38]			**Mode** Child group Parent group		
			**Setting** Not reported		
**Cool Kids Program- For Parents** Psychoeducation, management strategies, cognitive restructuring, coping	Parents attended group sessions with a clinical psychologist. Group sessions comprised of 8 topics: 1) psychoeducation about excessive anxiety in children; 2) parent management strategies for anxious children; 3) development of exposure hierarchies for children; 4) revision of exposure hierarchies; 5) cognitive restructuring for parents and children; 6) exposure for parents fears; 7) coping plans for children and 8) maintenance and relapse prevention. Parents were provided with a workbook and given homework to implement skills at home. A brief phone call was scheduled one month after the intervention to encourage maintenance.	*Therapist* Qualifications: Clinical psychological with experience in similar groups Relationship: Not reported	**Dosage** 12 hours *Parent group sessions*: 1 x 90 min session a week, for 8 weeks *Phone call*: One-off after 8-week intervention	Originally used for older children; modified for adults	● Psychoeducation, positive parenting skills ● In vivo exposure ● Cognitive restructuring
Kennedy, Rapee [39]			**Mode** Parent group		
			**Setting** Clinic		
**Cognitive bias modification training** Interpretation bias	Each session consisted of 10 ambiguous scenarios; each scenario consisted of 3 short sentences. The last word of the last sentence was missing one letter. In the positive training group, all final words made the story end positively. In the neutral group, all words made the story end in an irrelevant way. Children were asked to read each scenario and image themselves as the central character. Children pressed a button and the missing last word appeared with one letter missing. Children had to fill in the missing letter as quickly as possible. Children were asked a ‘yes’ or ‘no’ question that measured comprehension of the story. All children performed first training session in clinical with parent and trained research assistant. The remainder were completed at home.	*Self-directed* *Parent* Qualifications: NA Relationship: Parent *Therapist* Qualifications: Trained in intervention Relationships: Not reported	**Dosage** Total hours not reported *Training sessions*: Daily sessions for 14 days, preceded by practice session	Reading ability: Parent instructed to read sentences to child if needed	● Cognitive restructuring
Klein, Rapee [40]			**Mode** Child individual		
			**Setting** Clinic Home		
**UTalk- Interpersonal Psychotherapy Adolescent Skills Training** Social anxiety, depression, peer relationships, approaching other peers, coping with peer victimisation La Greca, Ehrenreich-May [41]	Initial individual sessions addressed key relationships with peers, education about four interpersonal problem areas: 1) role disputes; 2) role transitions; 3) interpersonal deficits; 4) role insecurity. The first three group sessions included psychoeducation, didactics on communication skills and role-play to practice communication skills. The following five group sessions includes didactics on communication and problem-solving skills, communication analysis to identify problematic communication, role-play and practice interpersonal skills. Interpersonal events in participants lives determined the group content of that week. The last two group sessions focused on reviewing skills and generalising to other scenarios. An individual mid-intervention session allowed for review of participants progress.	*Therapist* Qualifications: Postdoctoral or advanced graduate trainees in clinical psychology Relationships: Not reported	**Dosage** 17.25 hours *Individual sessions*: 3 x 45 sessions (2 x pre-group, 1 x mid intervention) *Group sessions*: 1 x 90-minute group weekly, for 10 weeks	Expanded to address social anxiety, including addressing peer relationships, psychoeducation, exposure practice and role insecurity	● Psychoeducation ● In vivo exposure
			**Mode** Child group Child individual		
			**Setting** Not reported		
**Second Life** Self-expression Lee [42]	Second Life is a virtual world in which participants can interaction. To maintain anonymity, participants create avatars and are only referred to by screenname. Two Second Life classes were held that the same time so no participants could be identified by other group members. Participants were assigned speaking topics through a chat function, which they presented to peers in the virtual Second Life class through microphone. Participants were allowed 15 lines of notes in preparation. Members sat in chairs while the speaker stook at a podium. After all participants spoke, they participated in a 10min question and answer session to discuss unclear points, good points and points to around. A moderator helped participants to manage environment and audio equipment	*Therapist* Qualifications: Not reported Relationship: Lead researcher	**Dosage** 480 minutes *Group sessions*: 2 x 40 sessions a week, for 6 weeks	Virtual space used to facilitate speaking sessions	● Peer mediation
			**Mode** Child group		
			**Setting** Online, virtual world		
			**Mode** Child group		
**Social Skills Training Facilitated Play (SST-FP)** Initiating/maintaining conversions, understanding/expressing feelings, emotion regulation, peer interaction	Group sessions involved 1) a 5 min free play session; 2) self-presentation speeches; 3) circle time and 4) leader-facilitated free play. Self-presentation speeches gave children opportunity to speak freely about a familiar topic (i.e. a new toy). Circle time involved didactic content, focusing on a set of social skills each week. These included initiating/maintaining interactions, understanding/expressing feelings and regulation of negative affect. Puppets and songs were used to convey content. During facilitated play, group leader guided participation, using prompting, modelling, encouragement and reinforcement of social skills taught.	*Therapist* Qualifications: Previous education and experience in early childhood education, trained by senior authors Relationship: Teacher of participants	**Dosage** 14 hours *Group sessions*: 2 x 60 min session a week, for 7 weeks	Adapted for young with young children in China	● Therapist modelling ● Behaviour modification
			**Mode** Child group		
Li, Coplan [43]			**Setting** School		
				
**Implosive, Counselling and Conditioning Approach** Eye contact, interest in communication with others, mixing socially Lowenstein [44]	Treatment consisted of group discussions between participants and psychologist, and individual sessions to establish a dialogue and increase non-verbal interaction skills. In individual sessions, participants were counselled on over-sensitivity and unrealistic fears. Counselling involved discovering early trauma that may contribute to shyness. Parents were encouraged to give children confidence and role play. Individual sessions involved conditioning eye contact through reinforcement. Group sessions involved peer-modelling assertive responses, and desensitisation procedures. Participants were encouraged to increase exposure by communication with dolls and increasing to unfamiliar children.	*Therapist* Qualifications: Psychologist, qualifications not reported Relationship: Not reported	**Dosage** Total hours not reported *Individual sessions*: Dosage of sessions not reported *Group discussions*: Dosage of sessions not reported *Duration of treatment*: 6 months	Therapists used ‘implosive’ approaches when needed. Where timidity was produced by punitive approach by parents, therapist would ‘force’ emotional reaction to gradually develop more positive reaction Participants provided with affection if showed lack of warmth Participants ‘forced’ to participate if showed immobility in activities If sensitive to loud noises, exposed to 30 mins of loud noise a day If particularly introverted, given extroverted roles in ‘drama therapy’	● In vivo exposure, ● Therapist modelling ● Psychoeducation ● Peer mediated
			**Mode** Child group Child individual		
			**Setting** Clinic		
**Cool Little Kids** Parental overprotection, avoidance	During each parent training session, topics were explained conceptually. Then practical skills and tool introduced to improve interactions with children, including cognitive restructuring and graded exposure. Practice tasks were assigned for parent to attempt with children at home.	*Therapists* Qualifications: Psychology lecturer and researcher, master’s degree in parenting education Relationship: Not reported	**Dosage** 9 hours *Group sessions*: 6 x 90-minute group session	None reported	● Psychoeducation ● Cognitive restructuring ● In vivo exposure
Luke, Chan [45]			**Mode** Parent individual **Setting** Kindergarten		
**Pyramid Program** Problem-solving, assertive communication, relaxation, emotional expression	Involves 10 to 12 children in the Pyramid Club. Week 1 involves naming and ownership of the club. Weeks 2–10 involve group sessions. Group sessions begin with circle time and a 20min art activity to encourage expression of feelings. Followed by cooperative games to facilitate problem-solving skills and role play, to learn about assertive responses. Participants then engage in laughing yoga, to aid relaxation, then conclude with a closing circle time.	*Therapist* Qualifications: Not reported Relationships: Not reported	**Dosage** 15 hours *Group sessions*: 1 x 90-minute sessions a week, for 10 weeks	None reported.	● Therapist modelling ● Peer mediation
McKenna, Cassidy [46]			**Mode** Child group		
			**Setting** School		
**INSIGHTS** Academic development, critical thinking, math, language, empathy, problem solving	Teachers and parents attending facilitated sessions based on the curriculum. One session included parents and teachers together, the rest were conducted separately. Parents learn to recognise temperament based on 4 typologies. Parents and teachers learn a ‘scaffold and stretch’ approach to challenging situations for their child. The classroom program was delivered concurrently. Curriculum involved puppets, workbooks and videotaped vignetted. Classroom sessions focused on empathy and problem-solving, using supports to learn 4 temperament typologies.	*Therapist* Qualifications: Graduate-level training in intervention Relationship: Not reported	**Dosage** Parent/teacher: 20 hours *Parent/teacher sessions*: 1 x 2-hour session a week, for 10 weeks Children: 7.5 hours *Classroom sessions*: 1 x 45 min session a week, for 10 weeks	None reported.	● Psychoeducation ● Therapist modelling
O’Connor, Cappella [47]			**Mode** Child group Parent group Teacher group		
			**Setting** School		
**Parent education program** Child temperament	Parents attend education sessions for management of children’s fears. Parents were educated about the nature of anxiety, techniques to tach their child to manage anxiety and how to manage their own anxiety. Parents were guided to reduce control, model more courageous coping, develop and use exposure hierarchies and use realistic self-talk.	*Therapist* Qualifications: Graduate student in clinical psychology Relationship: Not reported	**Dosage** 9 hours *Parent training*: 6 x 90-minute sessions, over 9 weeks	Modified for parents, previously designed for children	● Psychoeducation
Rapee and Jacobs [48], Rapee, Kennedy [49]			**Mode** Parent group		
			**Setting** Not reported		
**Cognitive-behavioural approach-based social skills training** Internalising behaviours	Sessions one involves familiarising the group, talking about importance of social skills and how to introduce self. Session two to nine involve skills training, including giving compliments, expression emotions, self-control, table manners, rejection methods, deal with criticism and collaboration at home. Session ten involved learning to accepted responsibility. Session eleven to fifteen involved explaining self-esteem, respect, conflict, family discipline and accepting rules. Session sixteen was a summary and final assessment. All sessions except the final involved practice of the skills/concepts taught.	Not reported.	**Dosage** 16 hours *Group sessions*: 2 x 60 min session per week, for 8 weeks	None reported.	● Social skills training
Sang and Tan [50]			**Mode** Child group		
			**Setting** Not reported		
**Group cognitive behavioural therapy** Relaxation, social skills	Focus of first two sessions were introduction of therapists and group members and setting treatment goals. The following three sessions were focused on progression muscle relaxation, training on subjective units of distress, anxiety in social situations, cognitive restructuring, and identification of automatic thoughts, and practice of differential relaxation. Sessions six to nine covered challenging automatic thoughts, gradual exposure, role play, social skills training and real-life exposure. Homework was given for each session. The last session involved activities and lessons learnt.	*Therapist* Qualifications: Not reported Relationship: Not reported *Co-therapist* Qualifications: Not reported Relationship: School counsellor	**Dosage** Total hours not reported *Group sessions*: 2 x session a week, for 5 weeks	None reported.	● Cognitive restructuring ● Therapist modelling ● Peer mediated ● Social skills training ● In vivo exposure
Umeh [51]			**Mode** Child group		
			**Setting** School		
**Emotional recognition training program** Emotion recognition, perception of happiness in others Rawdon, Murphy [52]	The training consists of a computerise task. Images of human faces with different emotions were presented to participants, in a random order. Images were present for 150 minutes. Participant was then asked to judge the emotion as either happy or disgusted. Each session consists of a baseline, training and test phase. Baseline and test phases consist of 45 trails, training phases consisted of 30 trials. During the training phase, participant received feedback (e.g. “Correct!”, “Incorrect!”) on their judgement of emotion. All participants were presented with the same stimuli. Control participants received feedback based on their baseline balance point (the point where participants shift from perceiving happiness to disgust in the stimulus). The intervention group received feedback when two faces closest to their baseline balance point at ‘disgust’ were then classified as ‘happy’ during training. This aimed to promote a shift of balance point and promote perception of happiness over disgust.	*Researcher* Qualifications: Not reported Relationship: Not reported	**Dosage** 1 hour *Group sessions*: 1 x 15-minute session, for 4 consecutive days	None reported.	● Cognitive restructuring
			**Mode** Child group		
			**Setting** School		


Descriptions of active intervention components reported in the included studies were reviewed and categorised. In terms of active intervention components, the studies used psychoeducation (*n* = 11), in-vivo exposure (*n* = 11), SST (*n* = 9), therapist modelling (*n* = 9), cognitive restructuring (*n* = 8), behaviour modification (*n* = 6), peer-mediation (*n* = 6) and video-modelling (*n* = 1). Across the studies, 12 used only one or two intervention components, while only five studies used a combination of 4 or more intervention components (see Table 3).

### Reported treatment outcomes

Across the included studies, significant reduction was reported for anxiety (*n* = 13), social phobia (*n* = 3), and internalising behaviours (i.e., withdrawal, avoidance, and isolation; *n* = 8). A significant improvement was found for play skills (*n* = 2) and aspects of social functioning (*n* = 8); social competence, social skills, social interaction, social adjustment, interpersonal skills, peer victimisation, perceived social support from peers, and pro-social behaviour. Further, four studies reported treatment gains to be maintained at follow-up periods between 6 months and 5 years (see Table 3).

### Meta-analysis

#### Effects of interventions

Twenty of the 25 studies were included in the meta-analysis. Five could not be included in the analysis as the data required were not reported [28,31,32,34,36]. Authors were contacted to collect the required data, but no responses were received.

Overall treatment effects were calculated for shyness interventions on within-group pre-post outcome measures. Sub-group analysis was conducted to compare the effect as a function of intervention characteristics: 1) setting (i.e., clinic, home, school, online or a combination); 2) mode of delivery (i.e., group interventions, individual interventions or both); 3) intervention focus (i.e., parent focused, child focused or both); and 4) rater of outcome measure (i.e., clinician-rated, parent-rated, self-report, teacher-rated or a combination).

Between groups analysis was also conducted to compare experimental groups post-interventions scores with those of the control groups. A further 3 studies were excluded from this analysis as they did not include control groups. The following four control condition types were included: 1) waitlist control groups where participants served as an untreated comparison group who eventually went on to receive the intervention; 2) control groups that received no intervention; 3) alternative treatment controls where participants received an intervention that did not have the approach of the intervention being tested; and 4) medication control groups, where participants received medication instead of the behavioural intervention.

#### Overall effect of shyness interventions

**Fig 2:**
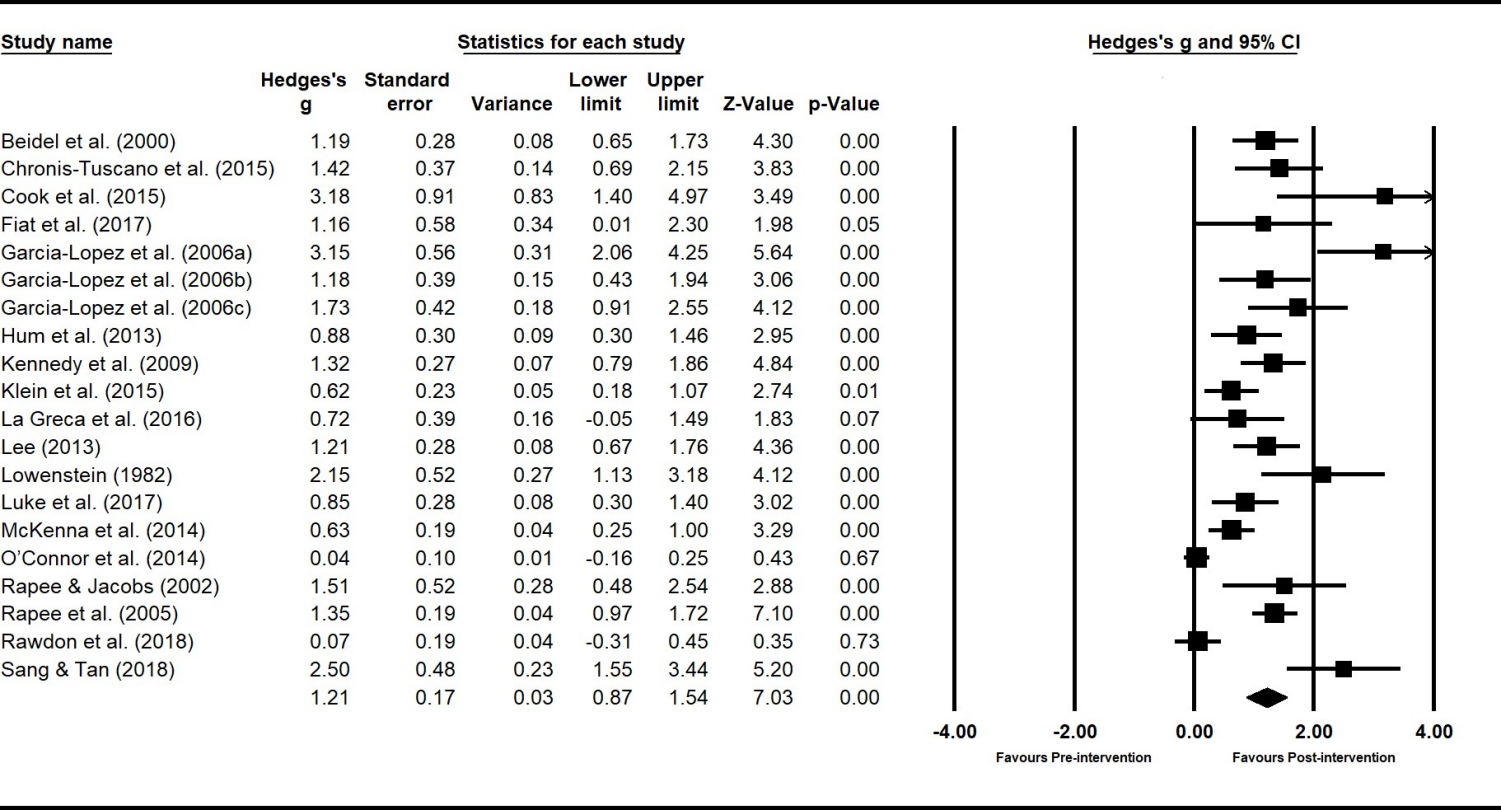
Within-group pre-post intervention meta-analysis.

Effect sizes ranged from 0.04 to 3.18 in the within-group pre-post intervention without groups analysis, as shown in Fig 2. Of the 20 studies included 75% (*n =* 15) produced a large effect size and 15% (*n =* 3) produced a moderate effect. An effect size of < 0.2 was measured in 10% (*n =* 2) of the studies. The overall intervention effect was large and statistically significant (z(20) = 7.03, *p* < .001, Hedge’s *g* = 1.21, 95% CI = 0.87–1.54). The between-study heterogeneity was significant *Q*(19) = 137.16, *p* < 0.001) and 86.2% of true variability (*I*^2^) could be explained by individual study characteristics.

#### Effect size as a function of intervention characteristics (within-group)

Table 4 shows the effect sizes of shyness interventions grouped by delivery setting, focus of the intervention, mode of delivery, and rater of outcome measures.

**Table 4:** Main results for within-groups sub-groups analysis.

Domains	Covariate	Hedge’s *g*	Standard error	95% Lower	95% Upper	Z value	2 tail *p* value
Setting	Clinic	1.38	0.13	1.12	1.63	10.50	0.001***
	Clinic and home	0.62	0.23	0.18	1.07	2.74	0.006**
	Online	1.21	0.28	0.66	0.75	4.36	0.001***
	School	1.03	0.26	0.52	1.55	3.91	0.001***
Focus	Child	1.34	0.23	0.89	1.78	5.93	0.001***
	Child and parent	0.73	0.44	-0.13	1.59	1.67	0.096
	Parent	1.24	0.13	0.98	1.50	9.42	0.001***
Mode	Group	1.02	0.23	0.57	1.47	4.42	0.001***
	Individual	1.05	0.32	0.41	1.69	3.25	0.001**
	Individual and group	1.60	0.30	1.01	1.50	5.29	0.001***
Rater	Clinician and parent	0.97	0.40	0.19	1.75	2.44	0.015*
	Clinician	1.16	0.22	0.72	1.60	5.17	0.001***
	Parent	2.50	0.48	1.55	3.44	5.20	0.001***
	Self-report	1.19	0.28	0.65	1.73	4.30	0.001***
	Teacher	1.39	0.61	0.18	2.59	2.26	0.024*

Note

* *p* < 0.050

** *p* < 0.010

*** *p* < 0.001.

*Setting*. Interventions that were delivered within a clinic demonstrated the largest effect size of those calculated as a function of setting (1.38), indicating a large, significant effect (*z*(9) = 10.50, *p* < .001, Hedge’s *g* = 1.38, 95% CI = 1.12–1.63). Interventions delivered online (*z*(1) = 4.36, *p* < .001, Hedge’s *g* = 1.21, 95% CI = 0.67–1.76) and those delivered in schools (*z*(9) = 3.91, *p* < .001, Hedge’s *g* = 1.03, 95% CI = 0.51–1.55) both produced a significant, large effect size. However, caution is needed when interpretation this results as only one study involved an online intervention. Interventions set in a combination of the home and a clinic produced the lowest effect size of all settings, showing a moderate, significant effect size (*z*(1) = 2.74, Hedge’s *g* = 0.62, 95% CI = 1.07–2.74). However this should be interpreted with caution as only one study used an intervention set in both a clinic and the home [40].

*Focus*. Interventions focused on the children alone produced the largest effect size of 1.33 of those calculated as a function of recipient of the intervention (*z*(13) = 5.93, *p* < .001, 95% CI = 0.89–1.78). Interventions that focused on both parents and children produced the lowest effect size, as demonstrated by a moderate but non-significant effect (*z*(3) = 1.67, Hedge’s *g* = 0.73, *p* = 0.1, 95% CI = -0.13–1.59).

*Mode of delivery*. Interventions that includes individual sessions, group sessions or both were all significant and large in effect. Those that utilised a combination of both individual and group sessions produced the largest effect (*z*(6) = 5.29, Hedge’s *g* = 1.6, *p* < .001, 95% CI = 0.88–1.5).

*Rater of outcome measures*. Interventions that used outcome measured rated by the children themselves, teachers, clinicians, parents or a combination of clinician and parents all produced large and significant effect sizes. Those that used measures completed by parents alone produced the largest effect size, however, this included only one study (*z*(1) = 5.2, Hedge’s *g* = 2.5, *p* < .001, 95% CI = 1.55–3.44). Those that used measures completed by clinicians and parents produced the lowest effect size, however, the effect size was still large and significant (*z*(2) = 2.44, Hedge’s *g* = 0.97, *p* < .05, 95% CI = 0.69–2.15).

#### Effect of shyness interventions compared with comparison groups (between-group)

**Fig 3:**
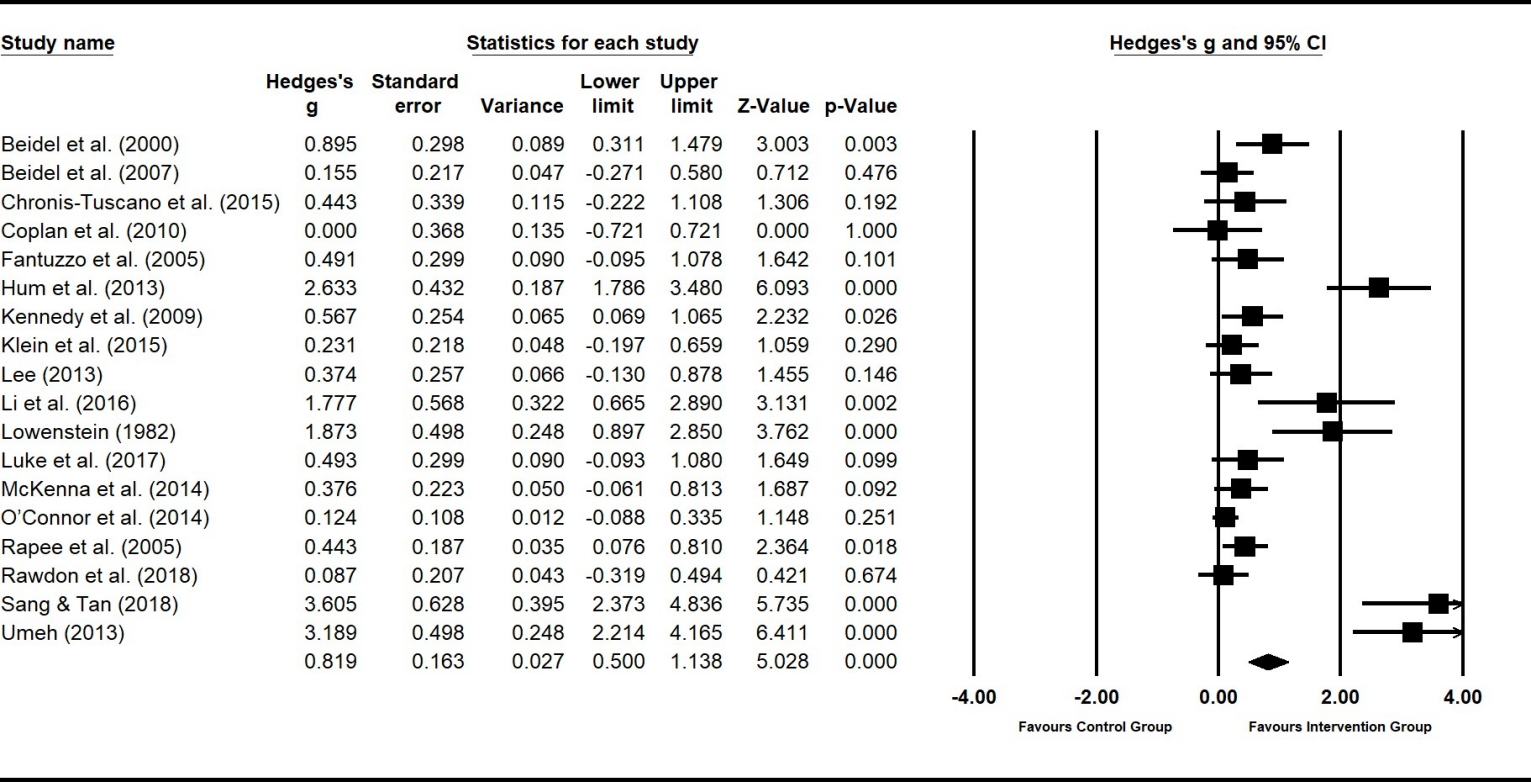
Between-group experimental vs. control intervention group meta-analysis.

As shown in Fig 3, shyness interventions for school-age children demonstrated a large, significant effect when compared to comparison groups (*z*(18) = 5.03, Hedge’s *g* = 0.82, *p* < .001, 95% CI = 0.5–1.14). Of the 18 studies included in the between-groups analysis, 33.3% (*n* = 6) produced a large effect size, 5.5% (*n* = 1) produced a moderate effect size, 38.8% (*n* = 7) produced a small effect size, and 22.2% (*n* = 4) produced a negligible effect size. The between-study heterogeneity was significant *Q*(17) = 113.84, *p* < 0.001) and 85.1% of true variability (*I*^2^) could be explained by individual study characteristics.

#### Effect size as a function of intervention characteristics (between-group)

Table 5 shows the effect sizes of shyness interventions grouped by delivery setting, focus of the intervention, mode of delivery, and rater of outcome measures when compared to control groups.

**Table 5:** Main results for between-groups sub-group analysis.

Domains	Covariate	Hedge’s *g*	Standard error	95% Lower	95% Upper	Z value	2 tail *p* value
Setting	Clinic	1.05	0.28	0.49	1.61	3.69	0.001***
	Clinic and home	0.23	0.22	-0.19	0.66	1.06	0.290
	Online	0.37	0.26	-0.13	0.88	1.46	0.146
	School	0.76	0.26	0.25	1.27	2.92	0.003**
Focus	Child	0.93	0.23	0.54	0.47	1.39	0.001***
	Child and parent	1.01	0.66	-0.28	2.30	1.54	0.123
	Parent	0.49	0.14	0.22	0.75	3.62	0.001***
Mode	Group	0.92	0.21	0.50	1.33	4.31	0.001***
	Individual	0.32	0.18	-0.02	0.67	1.83	0.068
	Individual and group	0.88	0.45	0.01	1.76	1.98	0.047*
Rater	Clinician and parent	0.29	0.18	-0.07	0.65	1.60	0.110
	Clinician	0.95	0.25	0.45	1.44	3.76	0.001***
	Parent	1.99	1.56	-1.05	5.05	1.29	0.199
	Self-report	0.50	0.37	-0.23	1.22	1.35	0.178
	Teacher	0.59	0.40	-0.19	1.37	1.48	0.139

Note

* *p* < 0.050

** *p* < 0.010

*** *p* < 0.001.

*Setting*. When compared to a control group, interventions delivered in a clinic produced the largest effect size of those calculated as a function of setting *z*(9) = 3.69, Hedge’s *g* = 1.05, *p* < .001, 95% CI = 0.5–1.61). Interventions delivered in a combination of the clinic and home, and those delivered online, produced small and non-significant effects. However, these only comprised of one study each. Interventions delivered in school produced a moderate, significant effect size (*z*(7) = 2.93, Hedge’s *g* = .76, *p* < .01, 95% CI = 0.25–1.27).

*Focus*. Interventions focusing on both children and their parents demonstrated a large but non-significant effect size when compared to control groups (*z*(3) = 1.54, Hedge’s *g* = 1.01, *p* = .123, 95% CI = -0.28–2.3). Those focusing on children alone demonstrated a large, significant effect size (*z*(12) = 3.95, Hedge’s *g* = .93, *p* < .001, 95% CI = 0.46–1.39). Interventions that focused on the parents alone produced a small but significant effect size (*z*(3) = 3.62, Hedge’s *g* = 0.49, *p* < .001, 95% CI = 0.22–0.75).

*Mode of delivery*. Interventions that used group sessions (*z*(13) = 4.31, Hedge’s *g* = .92, *p* < .001, 95% CI = 0.49–1.33) or a combination of individual and group sessions produced large effect sizes when compared to control groups (*z*(3) = 1.98, Hedge’s *g* = .88, *p* < .05, 95% CI = 0.1–1.75). Interventions using only individual sessions produced a small and non-significant effect when compared to control groups.

*Rater of outcome measure*. Interventions that used measures rated by parents demonstrated a large but non-significant effect size when compared to a control group, however, this included only 2 studies. Interventions that used measures rated by clinicians showed a large, significant effect size (*z*(9) = 3.76, Hedge’s *g* = .95, *p* < .001, 95% CI = .45–1.44).

**Fig 4:**
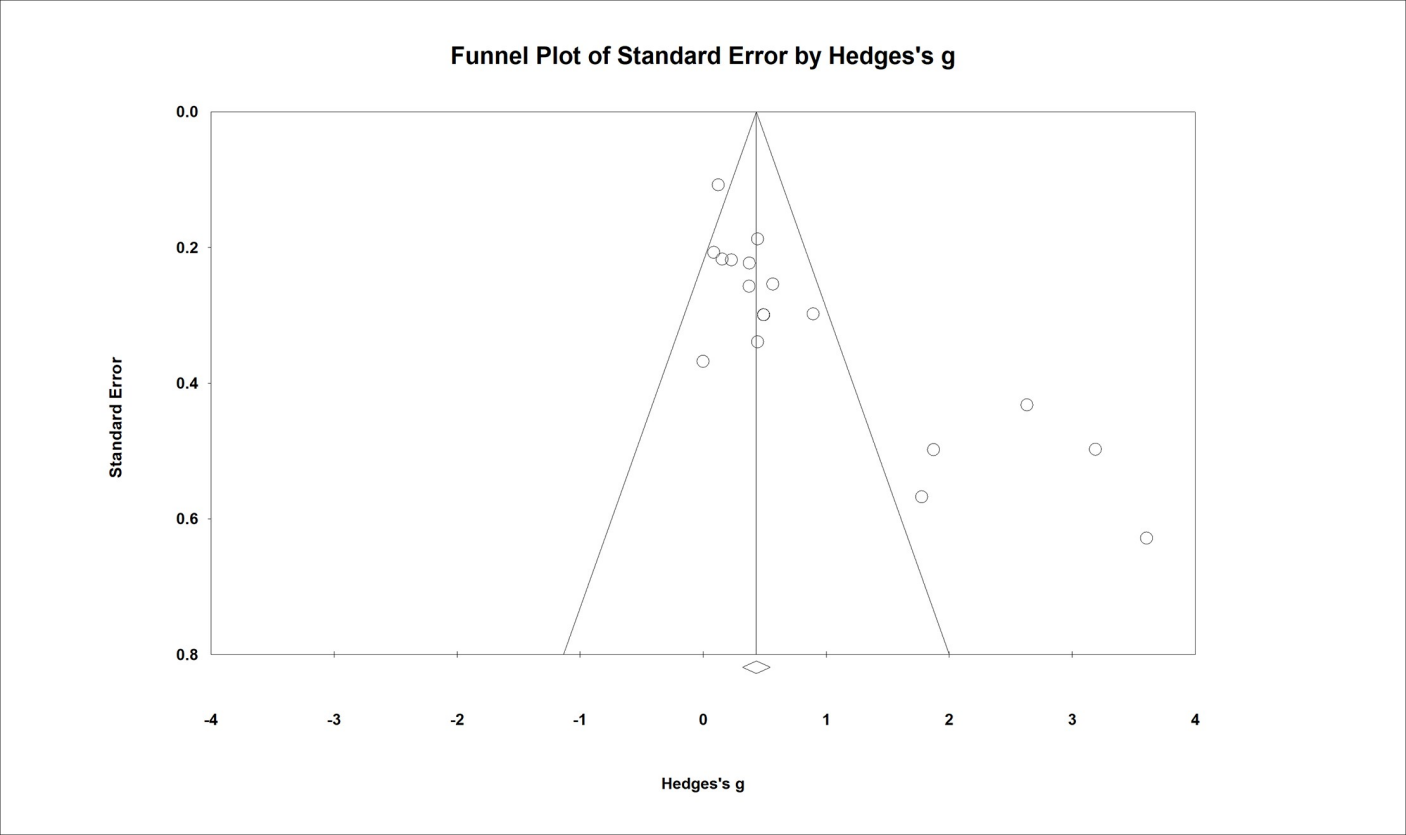
Funnel plot of standard error by Hedges’ g.

*Publication bias*. The Begg and Mazumdar rank correlation procedure produced a tau of 0.588 (two-tailed), indicating there is no evidence of publication bias. This finding was supported by Duval and Tweedie’s trim-and-fill procedure using the fixed-effect model; the point estimate for the combined studies is 0.433 (95% CI: 0.319, 0.546). Using trim and fill, these values are unchanged. Under the random-effects model the point estimate for the combined studies is 0.819 (95% CI: 0.499, 1.138). Using trim and fill, these values are unchanged. Both of these procedures indicate the absence of publication bias (see Fig 4 for funnel plot).

## Discussion

This study systematically identified available interventions for shy children and evaluated the effectiveness of these interventions in reducing psychosocial difficulties in school. Using systematic review and meta-analysis procedures, all study designs were included when identifying the available interventions. Both RCTs and quasi-experimental studies were included in the meta-analysis to broaden the scope and examine the effectiveness of all possible intervention studies for shy children.

The systematic review revealed that 25 studies met the inclusion criteria, comprised of 24 different interventions aiming to address shyness. All the included studies and the employed interventions were directed at school children, aged between six and twelve years. School is identified as a primary setting where shyness and its associated difficulties manifest or be noticed for the first time, as it is often a child’s first social environment away from parents. School often presents many different social situations for a child to navigate, such as classroom interactions, playgrounds and social cliques. Therefore, schools are suitable contexts for delivering ‘early’ intervention.

The results of the systematic review and meta-analysis support the association between intervention and reduction in shyness for this age group. As such, school-age may be an ideal developmental and social stage in life to target shyness to lessen the impact of shyness in school-age and later in life. However, the systematic review excluded any children outside of the age range, thus the systematic review cannot confirm that interventions at younger or older age groups, such as pre-school children, adolescence or young adults, are more or less effective. However, it is possible that shyness could be identified and addressed at earlier developmental stages or need intervention later into adolescence. A longitudinal study of fifth grade boys showed that, when children had better peer relationships, their shyness was more likely to decrease or remain stable over four years [53]. Those who were described as having poor peer relationships often increased in shyness.

Shy children may experience a wide range of difficulties in school that may impact their academic performance, social interactions and overall wellbeing [10]. A population-based, longitudinal study of children showed from ages 1.5 to 12.5 years, parent-reported shyness increased steadily over time [17]. Shyness that remained stable and increasing shyness also predicted poor social skills and higher levels of anxiety at the end of the follow-up [17]. The results of the current study suggest that by promoting protective factors and introducing intervention, shyness can change as a child matures into adolescence and young adulthood, but that without such protective factors, shyness can remain a hindrance. However, how adolescents and young adults experience shyness and the required composition of active intervention ingredients to affect change in shyness in this age group are not well understood. Further research is needed into the effectiveness of interventions for shyness for younger children and for adolescents, as well as long-term impacts of interventions into adulthood.

Most studies included interventions that were delivered in a school setting. The within-group meta-analysis revealed interventions in this setting showed a large effect in reducing shyness, which is consistent with extant literature regarding shyness in school. Historically, the classroom has been the setting for implementing shyness interventions, as teachers often notice and informally attempt to address reticent behaviour [20]. Such informal interventions often included tailoring material to accommodate a noticeably shy child, individualised support within the classroom, and using social learning strategies such as modelling and positive reinforcement [19,20,22]. Of the included interventions that were set in a school, none were set in the classroom, suggesting that extending interventions beyond the classroom can have a large impact on shyness. These school-based interventions often involved clinical methods such as social skills training, psychoeducation, and exposure. The interventions were often conducted in group sessions, based at the school, and involved activities such as play, modelling and reinforcement by the facilitator [28,29,32,34,36,43,46,47,51,52]. These methods have previously been criticised for not considering the social environment and peer interaction within which shyness manifest [8]. However, traditionally such methods have been confined to clinic settings and clinic-based interventions demonstrated the highest effect-sizes. However, the advantage of delivering interventions in a school setting, rather than a clinic setting, is the added value of ecological validity [54]. As such, the burden is less on school-based intervention, compared with clinic-based interventions, for treatment effects to generalise to a natural social context within which treatment strategies are applied [54,55]. The results of the current study show that, when such methods are used in a school-based setting and involve peers, the results can be effective in reducing shyness. This is consistent with recommendations by Mychailszyn, Cohen [23] and Crozier [1] that interventions should be age-appropriate, consider social development, and utilise school-wide programs that address all students, rather than targeted, clinic based interventions.

Findings from the within-group meta-analysis indicated that interventions that focused solely on the child produced the largest effect size when compared to other interventions that focused on parents alone or a combination of child and parent. Interventions focussing on both parents and children, often in the form of parent training and education, produced the lowest, non-significant effect size. This is contrary to previous recommendations that advocated for implementing interventions for shyness that involve both parents/carers as well as children themselves [23]. Wider literature regarding interventions for children with developmental disorders, such as autism spectrum disorder, have found that involved parent training and coaching alongside interventions for children is most effective in improving language and communication outcomes, compared to sole child or parent training [56]. This finding suggests that shyness may be unique to other conditions or disorders affecting social communication. This finding may be explained by how shyness develops. Early interactions between the child, their environment, parents and peers are believed to either promote or diminish the risk of later anxiety and shyness for the child [4,8,9]. It may be possible that a parent’s role in early development and supporting interactions between their child, environment, other adults, and peers may be more important than at an intervention level once shyness has developed. Therefore, parental involvement in shyness interventions may be more important when delivering interventions to children before they start school. The taxonomy of shyness proposes that shyness can stem from peer exclusion or different sources of fear within the child, including fear of novel social situations, fear in familiar situations and fear of perceived evaluation [7]. Given the results of the current meta-analysis, it may be possible that such internal (fear) and external (exclusion) sources of shyness are best addressed with the children, to resolve internal fears and promote inclusion with peers.

Overall, the between-groups meta-analysis revealed that all interventions of shyness demonstrated a large, significant effect size when compared to control groups of either no intervention, treatment as usual or medication interventions. When examining this effect as a function of setting, focus, mode, and rater of outcome, the results closely mirror that of the within-groups analysis. That is, clinic-based, child-focussed, and a combination of individual and group delivered interventions produced the largest effect sizes. Within-groups results should be interpreted with caution due to the lack of control group. However, the only difference between within-groups analyses and between-groups containing a control group was that, for between-group meta-analysis, group delivered interventions were slightly higher than a combination of individual and group delivered interventions.

The findings from this study builds on the evidence for effectiveness of interventions for shyness of school-aged children, by improving their social interactions with peer and reducing reticent behaviour. However, this review found no evidence of long-term benefits of reducing later development of social anxiety disorders or long-term impacts on educational and wellbeing outcomes. Further research with longitudinal follow-up is necessary to establish the long-term effectiveness of shyness interventions.

### Limitations

There was variation in how shyness was defined, conceptualised, and operationalised across the included studies. Some studies required a diagnosis of social phobia for inclusion into the intervention, whereas others relied on parent or teacher report of shy behaviours. This is reflective of definitional variation in the literature regarding shyness and limits the generalisability of the results found between studies. The children included in individual studies had a range of diagnoses that may have impacted the effectiveness of the included interventions. Further research is needed to examine effects of interventions for children with and without clinical levels of social anxiety and wider diagnoses. The current review focused on school-age children aged between six and twelve years. As such, no conclusions can be drawn about the effectiveness of interventions for younger children and adolescents or the long-term impacts of interventions. When examining settings of interventions, two categories only included one study each. Therefore, the results of these categories need to be interpreted with caution. This review was unable to ascertain which individual intervention components contributed most to the effectiveness of interventions. Further research is needed to isolate the active ingredients of the interventions and determine which contributes most to the effectiveness of interventions.

### Conclusion

Shyness impacts many school-aged children and can have lasting effects on peer interactions, wellbeing, psychosocial and academic achievement. The current study provides a comprehensive review of interventions for shyness, identifies the most commonly used strategies and intervention effectiveness. Of the 25 studies included in the review, most interventions were delivered weekly, to a group of children in a school-based setting. They employed strategies such as psychoeducation, exposure, modelling, cognitive restricting, and peer mediation to address shyness. Across all included studies, reductions were reported in anxiety, social phobia, and internalising behaviours. The meta-analysis revealed that clinic-based, child-focussed, and a combination of individual and group interventions wielded the most benefits in reducing shyness. However, school-based interventions also produced large effect-sizes and have ecological validity as an advantage. This systematic review and meta-analysis provide an evidence-based for the most effective interventions for shy children that must utilise clinical strategies, such as modelling and exposure, that should ideally be delivered in a school-based setting that facilitates interactions with peers.

## Supporting information

S1 Checklist PRISMA 2009 checklist. (DOC)
